# SEMA6B induces macrophage-mediated inflammation and hepatocyte apoptosis in hepatitis B virus-related acute-on-chronic liver failure

**DOI:** 10.7150/thno.97007

**Published:** 2024-08-19

**Authors:** Hui Yang, Qun Cai, Jiaojiao Xin, Xi Liang, Hozeifa Mohamed Hassan, Jiaxian Chen, Lulu He, Suwan Sun, Beibei Guo, Shiwen Ma, Bingqi Li, Xiaofei Zeng, Meiqian Hu, Peng Li, Jinjin Luo, Wen Hu, Heng Yao, Xingping Zhou, Yuheng Kong, Qiuzhi Wang, Xin Chen, Jing Jiang, Dongyan Shi, Jun Li

**Affiliations:** 1 State Key Laboratory for Diagnosis and Treatment of Infectious Diseases, National Clinical Research Center for Infectious Diseases, National Medical Center for Infectious Diseases, Collaborative Innovation Center for Diagnosis and Treatment of Infectious Diseases. The First Affiliated Hospital, Zhejiang University School of Medicine, Hangzhou, 310003, China.; 2 Department of Infectious Diseases and Liver Diseases, Ningbo Medical Center Lihuili Hospital, Affiliated Lihuili Hospital of Ningbo University, Ningbo, 315000, China.; 3 Precision Medicine Center, Taizhou Central Hospital (Taizhou University Hospital), Taizhou, 318000, China.; 4 Department of Infectious Diseases, Guizhou Provincial People's Hospital, Zunyi Medical University of Medicine, Guiyang, 550000, China.; 5 BioRigino Co., Ltd., Anji, 313300, China.; 6 Department of Pathology, The First Affiliated Hospital, Zhejiang University School of Medicine, Hangzhou, 310003, China.; 7 Institute of Pharmaceutical Biotechnology and the First Affiliated Hospital Department of Radiation Oncology, Zhejiang University School of Medicine, Hangzhou, 310058, China.; 8 Joint Institute for Genetics and Genome Medicine between Zhejiang University and University of Toronto, Zhejiang University, Hangzhou, 310003, China.

**Keywords:** acute-on-chronic liver failure, hepatitis B virus, biomarker, inflammation, apoptosis

## Abstract

**Rationale:** Patients with hepatitis B virus-related acute-on-chronic liver failure (HBV-ACLF) have a high short-term mortality rate. Semaphorin-6B (SEMA6B) plays a crucial role in the pathogenesis of HBV-ACLF, but its molecular basis remains unclear. This study aimed to elucidate the mechanisms of SEMA6B in HBV-ACLF progression.

**Methods:** A total of 321 subjects with HBV-ACLF, liver cirrhosis (LC), chronic hepatitis B (CHB), and normal controls (NC) from a prospective multicenter cohort were studied. 84 subjects (HBV-ACLF, n = 50; LC, n = 10; CHB, n = 10; NC, n = 14) among them underwent mRNA sequencing using peripheral blood mononuclear cells (PBMCs) to clarify the mechanisms of SEMA6B in HBV-ACLF. These mechanisms were validated through in vitro studies with hepatocytes and macrophages, as well as in vivo using SEMA6B knockout mice and mice treated with synthetic SEMA6B siRNA.

**Results:** Transcriptome analysis of PBMCs showed that SEMA6B was among the most differentially expressed genes when comparing patients with HBV-ACLF to those with LC, CHB, or NC. ROC analysis demonstrated the reliable diagnostic value of SEMA6B for HBV-ACLF in both the sequencing cohort and an external validation cohort (AUROC = 0.9788 and 0.9026, respectively). SEMA6B levels were significantly higher in the HBV-ACLF patients, especially in non-survivors, with high expression mainly observed in macrophages and hepatocytes in liver tissue. Genes significantly associated with highly expressed SEMA6B were enriched in inflammation and apoptosis pathways in HBV-ACLF non-survivors. Overexpression of SEMA6B in macrophages activated systemic inflammatory responses, while its overexpression in hepatocytes inhibited proliferation through G0/G1 cell cycle arrest and induced apoptosis. Knocking out SEMA6B rescued mice with liver failure by improving liver functions, reducing inflammatory responses, and decreasing hepatocyte apoptosis. Transcriptome analysis of liver tissue showed that SEMA6B knockout significantly ameliorated the liver failure signature, significantly downregulating inflammation-related pathways. Importantly, therapeutic delivery of synthetic SEMA6B siRNA also improved liver function, and reduced both inflammation and hepatocyte apoptosis in mice with liver failure.

**Conclusion:** SEMA6B, a potential diagnostic biomarker for HBV-ACLF, exacerbates liver failure through macrophage-mediated systemic inflammation and hepatocyte apoptosis. These findings highlight SEMA6B as a promising early treatment target for HBV-ACLF patients.

## Introduction

Acute-on-chronic liver failure (ACLF) is a life-threatening clinical syndrome that develops in patients with acute decompensation from liver cirrhosis or chronic liver disease, characterized by multiorgan failure and high short-term mortality rates^1^-^3^. The clinicopathological features of ACLF include hepatocyte necrosis, microvascular thrombosis, and inflammatory cell infiltration^4^. Hepatitis B virus-related ACLF (HBV-ACLF) progresses rapidly, with mortality rates remaining as high as 50% to 90% despite comprehensive treatment^2^. Therefore, understanding the pathogenesis of HBV-ACLF is critical for developing effective early diagnostic and prognostic strategies. Systemic inflammatory responses and immune metabolic disorders are considered important mechanisms in the pathogenesis of ACLF^5^, ^6^. A recent prospective multicenter study conducted by the Chinese Group on the Study of Severe Hepatitis B (COSSH) reported that innate immune activation, adaptive immune suppression, and metabolic imbalance caused by hepatitis B virus (HBV) reactivation were key mechanisms involved in the development and progression of HBV-ACLF. Among potential biomarkers associated with immune dysregulation in HBV-ACLF progression, Semaphorin-6B (SEMA6B) has been identified as a key molecule^6^.

Semaphorins (SEMAs) play critical roles in inflammation, angiogenesis, apoptosis, fibrosis, bone remodeling, and cell invasion^7^, ^8^. SEMA6B, the sixth member of the SEMA family, has shown significant correlations with the stage of liver fibrosis in hepatitis C virus-infected patients^9^. SEMA6B is also involved in inflammatory responses and serves as a biomarker for macrophage-specific genes^10^. Additionally, the level of SEMA6B in circulating monocytes has predictive value for cardiovascular disease^11^. In our previous study, we confirmed that SEMA6B can be a valuable biomarker for diagnosing HBV-ACLF^12^, but the specific mechanism of SEMA6B in HBV-ACLF progression remains unclear.

In this study, transcriptome sequencing was performed on peripheral blood mononuclear cells (PBMCs) from patients with HBV-ACLF, liver cirrhosis, chronic hepatitis B, or normal controls. SEMA6B was identified as a potential biomarker for diagnosing HBV-ACLF, and its molecular basis in the development and progression of HBV-ACLF was validated by in vitro and in vivo experiments.

## Methods

### Patients

A total of 321 patients from the prospective COSSH cohort were enrolled in the study. These comprised HBV-related acute-on-chronic liver failure (HBV-ACLF, n = 142), liver cirrhosis (LC, n = 60), and chronic hepatitis B (CHB, n = 60) patients. All PBMC samples were collected on the first day of hospitalization before any treatment was administered. Only liver transplantation-free patients were enrolled in this study. HBV-ACLF patients were diagnosed based on the COSSH-ACLF criteria and studied using stratified random sampling, based on the prevalence of ACLF grades in the COSSH cohort: ACLF-1 (60.6%), ACLF-2 (33.0%), and ACLF-3 (6.4%)^2^. LC patients were diagnosed based on pathology, endoscopy, radiology, laboratory examination, or clinical signs^6^. CHB patients were enrolled according to the 2009 American Association for the Study of Liver Diseases (AASLD) guidelines^13^. Healthy volunteers composed the normal control group (NC, n = 59). Cohort 1 (sequencing cohort: HBV-ACLF, n = 50; LC, n = 10; CHB, n = 10; NC, n = 14) and cohort 2 (external validation cohort: HBV-ACLF, n = 92; LC, n = 50; CHB, n = 50; NC, n = 45) were employed to confirm the role of SEMA6B in development and progression of HBV-ACLF.

### HBV-ACLF

HBV-ACLF was diagnosed based on the COSSH criteria^2^. This definition identified HBV-ACLF as a complicated syndrome with a high short-term mortality rate that develops in patients with HBV-related chronic liver disease, regardless of the presence of cirrhosis. It is characterized by acute deterioration of liver function and hepatic and/or extrahepatic organ failure. There are three grades of HBV-ACLF: ACLF-1, ACLF-2, and ACLF-3. ACLF-1 comprises four types of patients: (l) patients presenting with liver failure alone with an international normalized ratio (INR) > 1.5 and/or kidney dysfunction and/or hepatic encephalopathy (HE) grade I or II; (2) patients with kidney failure alone; (3) patients with a single organ failure in the coagulation, circulatory, or respiratory system and/or kidney and/or HE grade I or II; (4) patients with cerebral failure plus kidney dysfunction. ACLF-2 includes patients with two organ failures, while ACLF-3 comprises patients with three or more organ failures.

### LC, CHB and NC

LC was defined as patients with stable compensated cirrhosis. Cirrhosis was diagnosed based on previous liver biopsy results, clinical evidence of previous decompensation, laboratory tests, endoscopy (esophageal and gastric varices), and radiological imaging of portal hypertension and/or liver nodularity^6^. Patients with a history of decompensation (ascites, HE, upper gastrointestinal haemorrhage, bacterial infection) were excluded. Patients with CHB were enrolled according to the 2009 American Association for the Study of Liver Diseases (AASLD) guidelines^13^. Normal healthy volunteers (18-80 years old) were participants with a normal physical examination.

### mRNA sequencing and transcriptome analysis

Peripheral blood mononuclear cells (PBMCs) were isolated from 84 subjects (cohort 1) using a Ficoll-Paque density gradient. Total RNA was extracted from PBMCs or mouse liver tissues and purified using TRIzol reagent following the manufacturer's instructions. mRNA sequencing was conducted using the TruSeq RNA LT Sample Prep Kit v2 (Illumina). Fastaq software was utilized to filter out reads containing adaptor contaminants, low-quality bases, and undetermined bases with default parameters. Expression levels of mRNAs were quantified using StringTie by calculating the fragments per kilobase per million reads (FPKM). Principal component analysis was performed using the R package “prcomp” Differentially expressed genes were identified using DESeq.2 in R, selecting genes with |log2 (fold change) | > 1 and adjusted p-value < 0.05. Gene set enrichment analysis (GSEA) was conducted using pre-ranked gene lists, focusing on biological processes using Gene Ontology (GO) biological process and Kyoto Encyclopedia of Genes and Genomes (KEGG) pathway gene sets from the Molecular Signatures Database (MSigDB). A significance value of p < 0.05 was applied for GSEA. The same analytical protocol was applied to the mouse liver tissues.

### Cell culture and treatment

The mouse macrophage cell line (RAW264.7) (Cell Bank of the Chinese Academy of Science) was cultured in Dulbecco's modified Eagle's medium (DMEM) (Gibco) supplemented with 10% fetal bovine serum (FBS) (Gibco) and 1% antibiotics (penicillin and streptomycin) (Gibco). The mouse hepatocyte cell line (AML12) (Cell Bank of the Chinese Academy of Science) and human hepatocyte cell line (HepaRG) (American Type Culture Collection) were cultured in DMEM with dexamethasone (40 ng/mL), ITS Liquid Media Supplement (1%) (Gibco), 10% FBS, and 1% antibiotics. The human monocyte cell line (THP1) (Cell Bank of the Chinese Academy of Science) was cultured in Roswell Park Memorial Institute (RPMI) 1640 (Gibco) supplemented with 10-20% FBS. THP1 derived macrophages were induced by phorbol 12-myristate 13-acetate (PMA, 100 ng/mL) (Sigma) for 24 hours. All cells were incubated in a 37°C, 5% CO2 incubator. To evaluate SEMA6B-mediated inflammatory responses. Macrophages (RAW264.7 and THP1) were stimulated with lipopolysaccharide (LPS) (100 ng/mL) for 6, 12, and 24 hours. Cell culture supernatants were collected post-stimulation. Hepatocytes (AML12 and HepaRG) were stimulated with LPS (100 ng/mL) for 24, 48, and 72 hours. The culture medium was replaced with DMEM supplemented with 2% FBS before stimulation.

To clarify the detailed mechanisms of SEMA6B in macrophage and hepatocytes. RAW264.7-SEMA6B knockdown (SEMA6B^KD^) cells were generated using CRISPR/Cas9 technology. Single guide RNAs (sgRNAs) targeting SEMA6B were cloned into the lenti-CRISPRv2 vector. Viral stocks were produced by co-transfecting modified plasmids with pMD2.G and psPAX2 into 293T cells. RAW264.7 cells were infected with virus collected from the supernatant and treated with puromycin for 1 week to select stable knockdown cells. THP1-SEMA6B knockdown and HepaRG-SEMA6B knockdown cells were constructed using human siRNA targeting SEMA6B (20 nmol/L) (GenePharma) and Lipofectamine RNAi Max (Thermo Fisher Scientific). SEMA6B overexpression (SEMA6B^OE^) was achieved by transfecting RAW264.7 and AML12 cells with lentiviruses produced by GenePharma. Stable overexpressing cell lines (RAW264.7-SEMA6B^OE^ and AML12-SEMA6B^OE^) were established, and knockdown and overexpression efficiencies were validated using western blot analysis. The primer pairs of sgRNA and siRNA were listed in Table S4.

### Proteomic sample preparation and analysis

Proteins were extracted from RAW264.7-SEMA6B^WT^ and RAW264.7-SEMA6B^OE^ cells. The total protein concentration was quantified by a BCA protein assay kit. Proteins interacting with SEMA6B were pulled down using an antibody against SEMA6B (Santa Cruz Biotechnology) and IgG from the same host. These proteins were reduced with 5 mM dithiothreitol at room temperature for 30 minutes and subsequently alkylated in the dark with 15 mM iodoacetamide for 30 minutes. After alkylation, the proteins were cleaned by acetone precipitation and resuspended in 50 mM ammonium bicarbonate. The samples were subjected to trypsin digestion (enzyme/protein ratio of 1:50) overnight at 37°C. Protein profiles were analyzed by liquid chromatography-mass spectrometry/mass spectrometry (LC-MS/MS) using a U3000 nanoUPLC system. The acquired raw files from LC-MS/MS were analyzed by MaxQuant (version 1.4.1.2) and searched against concatenated UniProt protein databases.

### Detection of multiple cytokines

To validate the role of SEMA6B in inflammatory response, cell culture supernatants were collected. The inflammatory cytokines were detected using the Mouse/Human Inflammation Array 1 (QAM-INF-1, FAM-INF-1, FAH-INF-1) according to the manufacturer's instructions (Ray Biotech).

### Flow cytometry

For the cell cycle assay, a Cell Cycle Staining Kit (Yeasen) was used according to the manufacturer's protocol. Briefly, AML12/HepaRG cell lines and primary mouse hepatocytes (PMHs) were harvested and fixed in 70% ethanol overnight at 4°C, then treated with RNase A and propidium iodide (PI) at 37°C in the dark for 30 minutes. For the cell apoptosis assay, AML12/HepaRG cell lines and PMHs were incubated with Annexin V-FITC or PI at room temperature in the dark for 15 minutes, following the instructions of the Annexin V/PI apoptosis kit (Yeasen).

### CCK8 assay

Cell proliferation was assessed using the Cell Counting Kit-8 (MCE) following the manufacturer's instructions.

### Animal studies

Male wild-type (WT) and SEMA6B knockout (KO) C57BL/6 mice aged 6-7 weeks (25-30 g) were obtained from GemPharmatech. Mice were housed in a standard specific pathogen-free (SPF) environment with a 12-hour light/dark cycle, temperature- and humidity-controlled conditions, and free access to water and food. All animal procedures were conducted in accordance with the criteria described in the 'Guide for the Care and Use of Laboratory Animals'. Isoflurane was used to anesthesia.

WT mice were randomly divided into two groups and received intraperitoneal injections of 5'-Cholesterol-SEMA6B siRNA (5 mg/kg) or negative control prior to 42 hours of LPS/D-gal infection. siRNA (μg) and in vivo RNA transfection reagent (μL) (Entranster-in vivo; Engreen Biosystem) (2:1) were mixed in a 5% sucrose solution according to the manufacturer's instructions.

Mice were injected intraperitoneally with a combination of D-gal (800 mg/kg) and LPS (50 μg/kg) dissolved in sterile saline (0.5 mL per mouse) and sacrificed 5.5 hours after injection. Serum samples were collected, and centrifuged at 3500 rpm for 10 minutes at 4°C, and stored at -80°C for biochemistry and cytokine analysis. Fresh liver tissues were fixed in 10% formalin for histological assessment and flash-frozen in liquid nitrogen for RNA and protein analysis.

### Isolation and culture of primary macrophages and hepatocytes

Primary mouse macrophage cells (bone marrow-derived macrophages, BMDMs) were prepared by collecting the bone marrow cells from the femurs of WT and SEMA6B^KO^ mice. BMDMs were cultured in DMEM supplemented with recombinant mouse granulocyte-macrophage colony-stimulating factor (GM-CSF, 50 ng/mL) (MCE) and 20% FBS. A two-step collagenase perfusion method^14^ was employed to isolate primary hepatocytes from WT and SEMA6B^KO^ mice. Primary mouse hepatocytes (PMHs) were cultured in DMEM supplemented with dexamethasone (40 ng/mL), 1% ITS Liquid Media Supplement, 10% FBS and 1% antibiotics. Both BMDMs and PMHs were stimulated with LPS (100 ng/mL).

### Survival curve analysis

WT and SEMA6B^KO^ mice were monitored for 24 hours following LPS/D-gal injection. An experienced expert in animal studies assessed mouse survival. Survival curve analysis was conducted using the nonparametric Mantel-Cox test to compare the survival rates.

### Biochemical function evaluation

Liver function was assessed by measuring classical biomarkers, including alanine aminotransferase (ALT), aspartate aminotransferase (AST), total bile acid (TBA), and total bilirubin (TBil), using an automatic clinical chemistry analyzer.

### ELISA

To validate the cytokine levels, we assessed two representative cytokines (IL-6 and TNF-α) in mouse serum by ELISA, following the manufacturer's instructions (Ray Biotech).

### TUNEL assay

Apoptosis was detected with a TUNEL assay kit (Abcam). The number of TUNEL-positive cells was quantified by counting five randomly selected high-power fields in each section, with 3-4 sections analyzed per group.

### Real-time quantitative reverse transcription PCR (qRT-PCR), western blotting and immunohistochemistry (IHC)

Detailed protocols for qRT-PCR, western blot, H&E staining, and IHC are provided in the supplemental methods. The antibodies and primer pairs were listed in Table S3 and Table S4.

### Statistical analysis

The results of all measurements are presented as the mean ± SEM, median (p25, p75), or number (percentage). Data analysis was conducted using SPSS V.25 (SPSS) and GraphPad Prism V.8.0. Student's t test and the Mann-Whitney U test were performed for two groups of continuous data. The Kruskal-Wallis test was used for multigroup comparisons of continuous variables. The χ2 test and Fisher's exact test were applied to compare categorical variables. Significance levels are denoted as follows: *p < 0.05; **p < 0.01; ***p < 0.001; ****p < 0.0001.

## Results

### Patients and Clinical characteristics

The demographic and clinical characteristics of all 321 subjects enrolled in this study were summarized in Table 1. HBV DNA levels were significantly higher in the HBV-ACLF group compared to the LC/CHB groups. Laboratory indicators, including alanine aminotransferase (ALT), total bilirubin (TBil), and international normalized ratio (INR), were significantly higher in the HBV-ACLF group compared to the LC/CHB/NC groups. The most frequent organ failure in the HBV-ACLF group was liver failure (95.1%), followed by coagulation failure (38.7%), brain failure (5.6%), and kidney failure (3.5%). Liver transplant-free mortality at 28 and 90 days was 30.4% and 42.9%, respectively, in the HBV-ACLF group. Among the 84 subjects in the sequencing cohort, one sample in the LC group did not meet the minimum qualification criteria (>20 M) and was excluded from further analysis.

### SEMA6B as a diagnostic biomarker for HBV-ACLF

**Figure 1:**
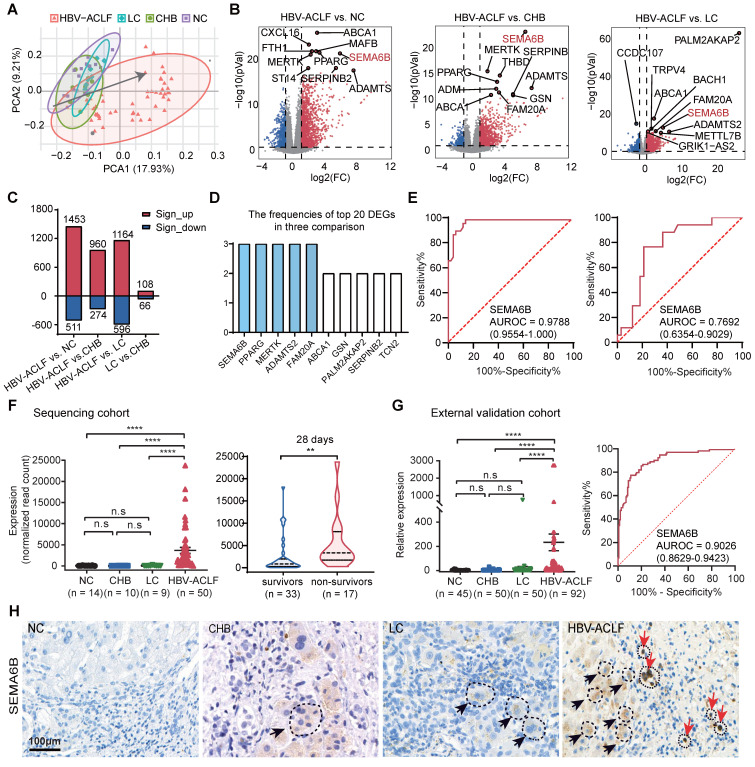
** Identification of SEMA6B as a potential diagnostic biomarker for HBV-ACLF progression.** (A) PCA of subjects in the HBV-ACLF, LC, CHB, and NC groups; the gray line indicates the direction of disease progression. (B) Volcano plot showing the top 10 significant DEGs from the pairwise comparisons of HBV-ACLF versus LC, HBV-ACLF versus CHB, and HBV-ACLF versus NC. Significantly differentially expressed genes (|log2-fold change| > 1; adjusted p-value < 0.05) are shown in red (upregulated) and blue (downregulated). (C) The number of significant DEGs in four pairwise comparisons. Red and blue columns represent the significantly upregulated and downregulated DEGs, respectively. (D) Frequencies of the top 10 genes among the top 20 significant DEGs in the three comparisons: HBV-ACLF versus LC, HBV-ACLF versus CHB, and HBV-ACLF versus NC. (E) ROC curves of SEMA6B levels for distinguishing patients with HBV-ACLF from those with LC, CHB, and normal controls (left), and for distinguishing 28-day HBV-ACLF survivors from non-survivors (right). (F) SEMA6B levels in patients from the HBV-ACLF, LC, CHB and NC groups (left) (****p < 0.0001, ns: not significant), and in 28-day HBV-ACLF survivors versus non-survivors (right) (**p < 0.01) in the sequencing cohort. (G) SEMA6B levels in patients with the HBV-ACLF, LC, CHB, and NC groups (left) (****p < 0.0001, ns: not significant), and ROC curves of SEMA6B expression levels for distinguishing patients with HBV-ACLF from those with LC, CHB, and normal controls in the external validation cohort. (H) Immunohistochemical staining for SEMA6B in patients from the HBV-ACLF, LC, CHB, and NC groups. Three subjects per group. Black and red arrows indicate hepatocyte-like and immune cell-like positive staining, respectively (bar = 100 μm). ACLF, acute-on-chronic liver failure; LC, liver cirrhosis; CHB, chronic hepatitis B; NC, normal control; PCA, principal component analysis; DEGs, differentially expressed genes.

Principal component analysis (PCA) of PBMC transcriptomic data revealed that distinct clustering of patients with HBV-ACLF, separate from those with LC, CHB, and NC (Figure 1A). The distribution along the gray line in Figure 1A indicated the progression of HBV-ACLF among the four groups (NC/CHB/LC/HBV-ACLF). Pairwise differential expression analysis highlighted significant differences in gene expression signatures between the HBV-ACLF group and other groups (Figure 1B-C). Specifically, we identified 1453 significantly upregulated and 511 downregulated differentially expressed genes (DEGs) in the HBV-ACLF group compared to the NC group, 960 upregulated and 274 downregulated DEGs compared to the CHB group, and 1164 upregulated and 596 downregulated DEGs compared to the LC group (Figure 1B-C). Comparisons between the CHB and LC groups showed relatively fewer DEGs (Figure 1C, Figure S1).

Next, we analyzed the frequencies of the top 20 differentially expressed genes (DEGs) in the HBV-ACLF versus NC/CHB/LC groups, identifying SEMA6B, PPARG, MERTK, ADAMTS2, and FAM20A as the top 5 significantly upregulated genes in the HBV-ACLF group (Figure 1D). ROC analysis demonstrated their discriminative accuracy between HBV-ACLF and non-ACLF groups (LC, CHB, and NC), as well as between HBV-ACLF non-survivors and survivors (Figure 1E, Figure S2). Notably, SEMA6B exhibited the reliable predictive value for identifying HBV-ACLF patients (AUROC = 0.9788) compared with LC, CHB, and NC groups, with distinguishing HBV-ACLF non-survivors from HBV-ACLF survivors (AUROC = 0.7692) (Figure 1E). SEMA6B levels were significantly elevated in the HBV-ACLF group compared to the LC/CHB/NC groups, particularly in HBV-ACLF non-survivors within the sequencing cohort (Figure 1F), findings that were validated in an external cohort using qRT‒PCR and immunohistochemical staining (Figure 1G-H, Figure S3). Based on these results, SEMA6B levels did not differ significantly between LC and CHB groups, prompting their categorization as chronic liver disease (CLD) for subsequent analyses. Moreover, SEMA6B exhibited a diagnostic accuracy of 0.9026 for predicting HBV-ACLF in the external validation cohort (Figure 1G). These findings underscore SEMA6B as a robust diagnostic biomarker for HBV-ACLF.

### SEMA6B associated with disease severity and inflammation in HBV-ACLF progression

**Figure 2:**
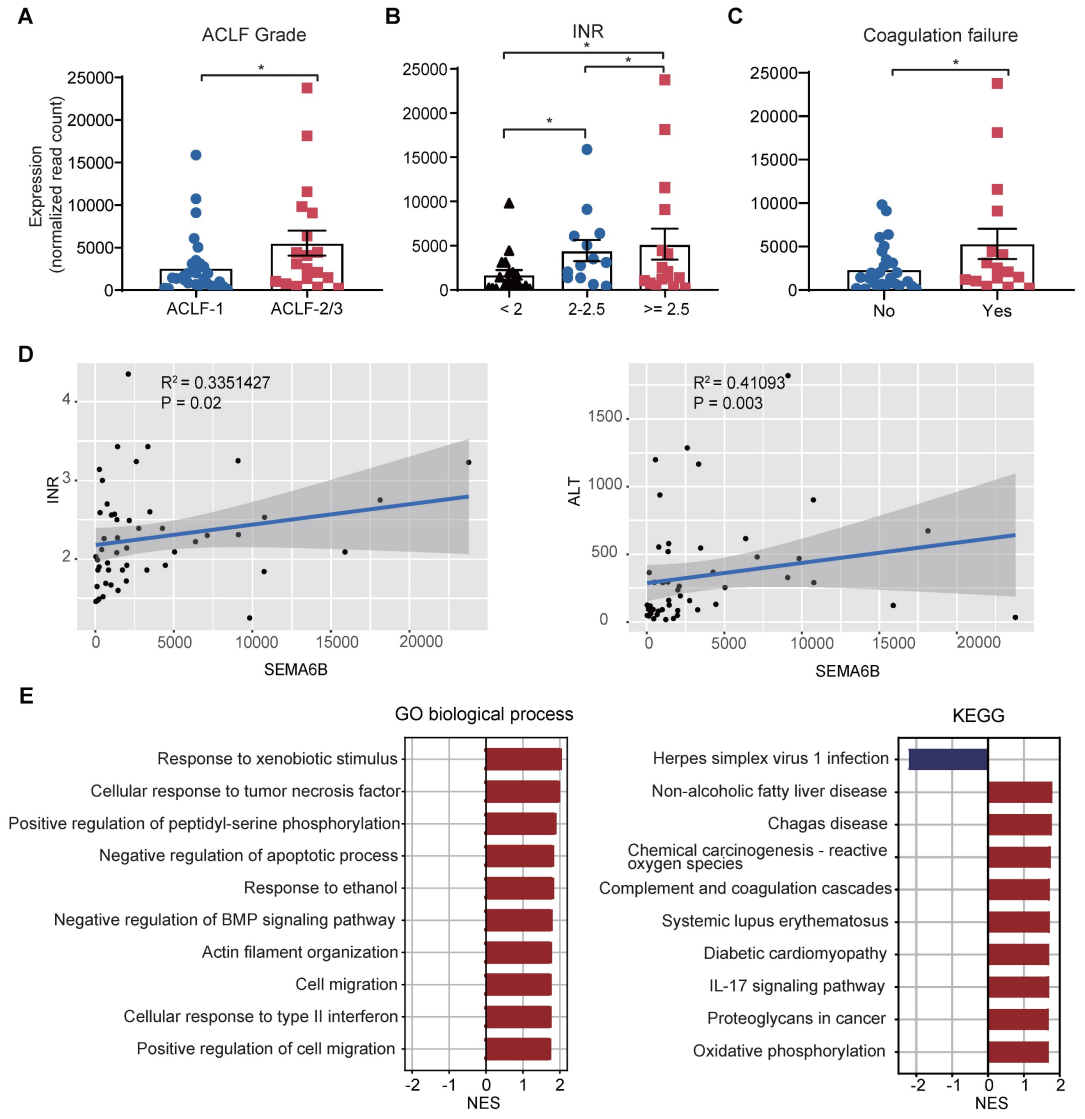
** SEMA6B expression levels are strongly associated with ACLF disease severity and inflammation.** (A) SEMA6B expression levels stratified by ACLF grade. *p < 0.05. (B) SEMA6B expression levels stratified by INR. *p < 0.05. (C) SEMA6B expression levels stratified by coagulation failure status. *p < 0.05. (D) Correlation of SEMA6B expression levels with clinical indicators (INR, ALT) in the ACLF group. Spearman's correlation coefficient (r) and the p-value of each correlation are annotated. (E) Gene set enrichment analysis results using the gene list ranked by Pearson's correlation coefficient values associated with SEMA6B expression. The top 10 significant Gene Ontology (GO) biological processes and KEGG pathways are identified. ACLF, acute-on-chronic liver failure; INR, international normalized ratio; ALT, alanine aminotransferase; GO, Gene Ontology; NES, normalized enrichment score; KEGG, Kyoto Encyclopedia of Genes and Genomes.

We investigated the relationship between SEMA6B levels and key clinical indicators, as well as the biological processes involving SEMA6B in HBV-ACLF progression within the sequencing cohort. SEMA6B levels were found to significantly increase with ACLF grade (Figure 2A) and across three INR level groups (INR < 2, 2 ≤ INR < 2.5, INR ≥ 2.5) (Figure 2B). Significant differences in SEMA6B levels were observed between patients with and without coagulation failure (p < 0.05) (Figure 2C). Moreover, SEMA6B expression levels correlated significantly with key clinical indicators such as INR (R = 0.335, p = 0.02) and ALT (R = 0.411, p = 0.003) (Figure 2D, Table S1). Gene set enrichment analysis (GSEA), using Pearson's correlation coefficient values ranked by SEMA6B expression, revealed a significant positive correlation between SEMA6B levels and pathways associated with poor outcomes in HBV-ACLF. These pathways included inflammation-related processes such as IL-17 signaling, cellular response to tumor necrosis factor/type II interferon, response to xenobiotic stimulus, and complement and coagulation cascades (Figure 2E). These findings indicate that elevated SEMA6B levels in HBV-ACLF patients are closely associated with disease severity and heightened inflammatory responses.

### SEMA6B highly expressed in hepatocytes and macrophages of HBV-ACLF patients

To identify the cell types expressing SEMA6B during HBV-ACLF progression, we analyzed publicly available RNA-seq data and performed immunofluorescence staining assays. Analysis of PBMC RNA-seq data from healthy donors revealed high SEMA6B expression in myeloid cells, including neutrophils, classical monocytes, and myeloid DCs (Figure S4). Single-cell RNA-seq data from healthy liver tissues indicated SEMA6B expression in hepatocytes, macrophages, and endothelial cells (Figure S5).

**Figure 3:**
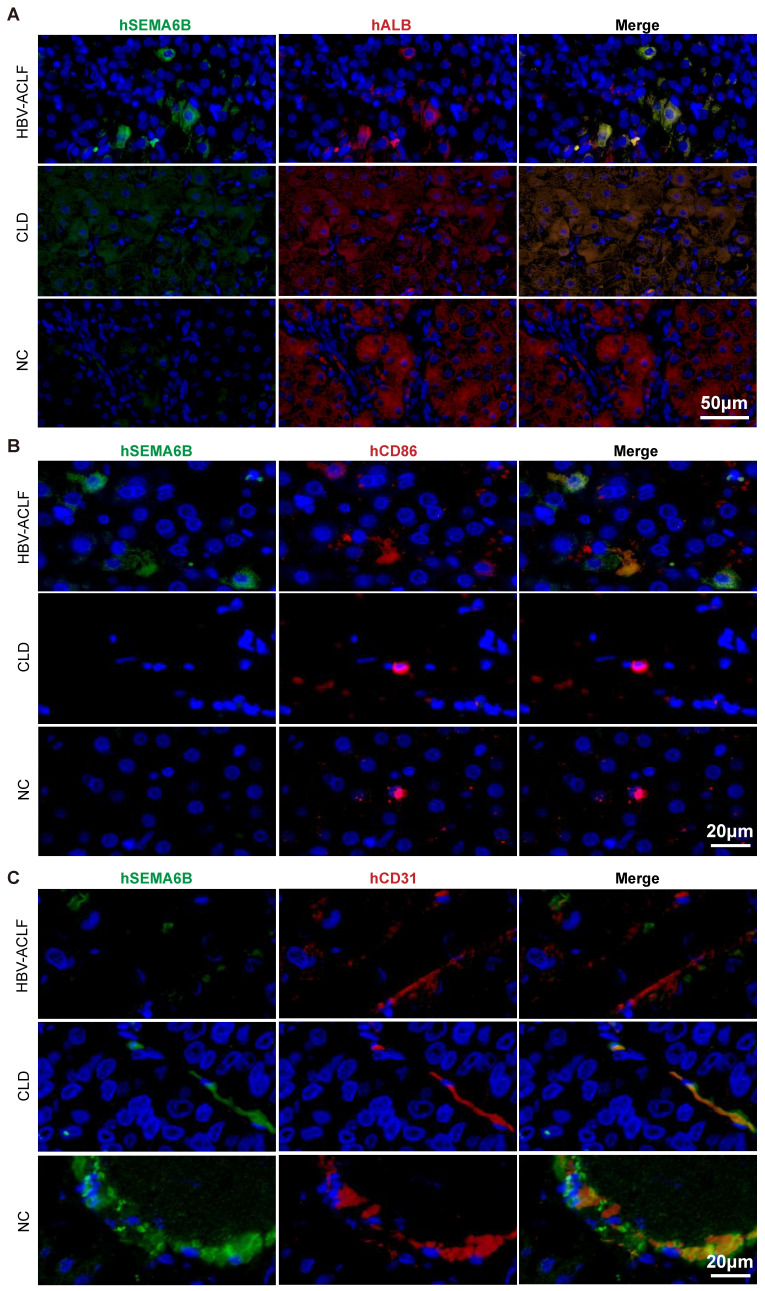
** Immunofluorescence staining of human liver tissues depicting the cell types that highly express SEMA6B in HBV-ACLF patients.** Co-immunofluorescence staining for hSEMA6B (green) (A) a hepatocyte marker (hALB; red), (B) a macrophage marker (hCD86; red), and (C) an endothelial cell marker (hCD31; red) in liver tissues from patients with HBV-ACLF, CLD and NC. Nuclei stained with DAPI (blue). Scale bars: (A) 50 μm; (B) 20 μm; (C) 20 μm. ALB: albumin; HBV-ACLF, hepatitis virus B related acute-on-chronic liver failure; CLD: chronic liver disease; NC: normal control.

Immunofluorescence staining further confirmed these findings. SEMA6B was highly expressed in albumin (ALB)-positive hepatocytes in liver tissues of the HBV-ACLF group, with lower expression in the chronic liver disease (CLD) group and no expression in the NC group (Figure 3A). Additionally, CD86 and SEMA6B double-positive macrophages were observed only in the HBV-ACLF group, but not in the CLD and NC groups (Figure 3B). SEMA6B and CD31 double-positive endothelial cells were absent in the HBV-ACLF group but present in the CLD and NC groups (Figure 3C). These findings provide strong evidence that SEMA6B is highly expressed in hepatocytes and macrophages within liver tissues of HBV-ACLF patients, highlighting its potential role in the pathogenesis of HBV-ACLF.

### SEMA6B overexpression activates macrophage mediated inflammatory responses and induces hepatocyte apoptosis

**Figure 4:**
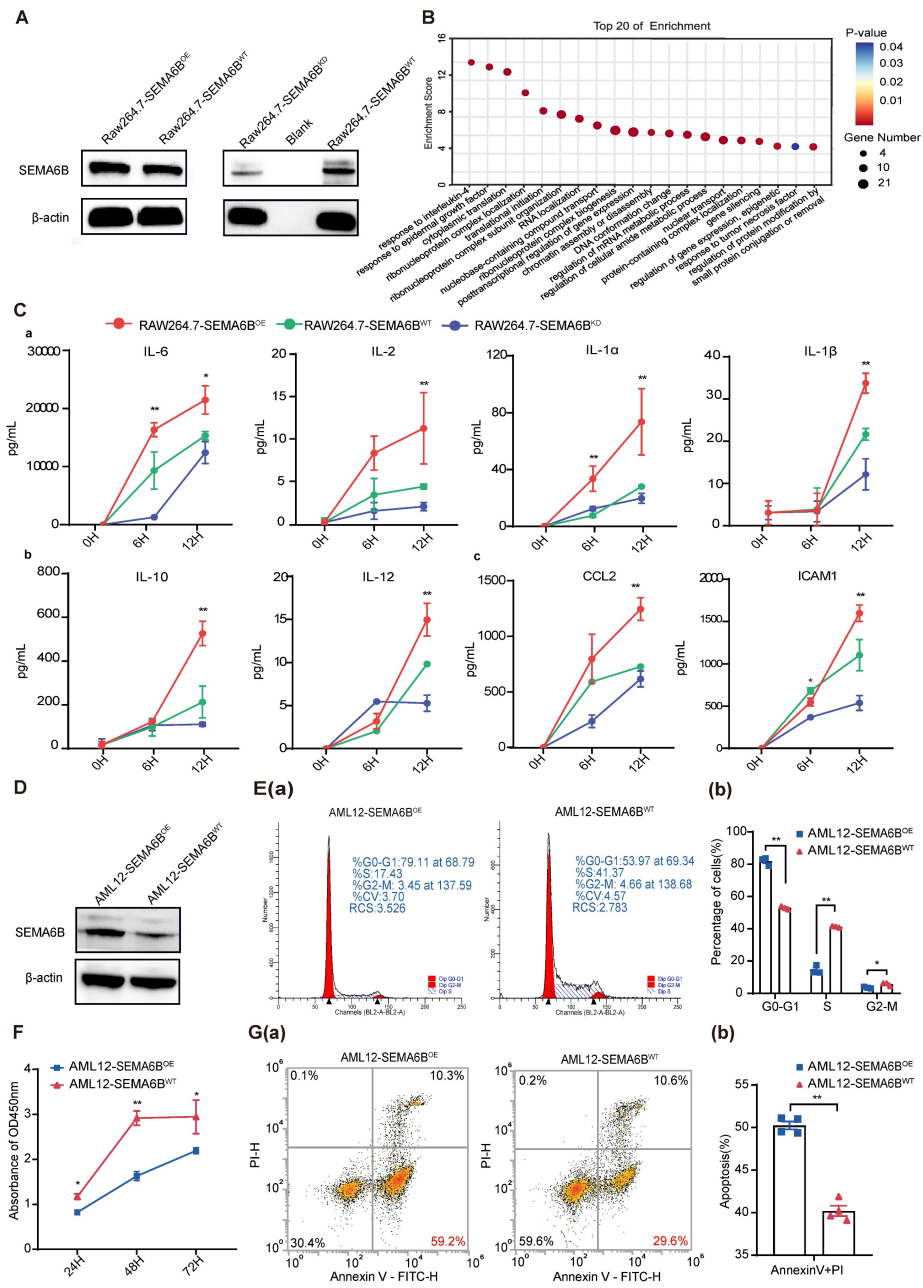
** Evidence for the functions of SEMA6B in macrophages and hepatocytes.** (A) Western blot showing SEMA6B expression levels in the RAW264.7-SEMA6B^OE^, RAW264.7-SEMA6B^KD^, and RAW264.7-SEMA6B^WT^ groups. (B) Gene Ontology analysis of proteins interacting with SEMA6B, highlighting the top 20 significant biological processes. (C) Concentrations of inflammatory cytokines in the supernatants of RAW264.7-SEMA6B^OE^ (red), RAW264.7-SEMA6B^KD^ (blue), and RAW264.7-SEMA6B^WT^ (green) cells at 0 h, 6 h, and 12 h after LPS stimulation. *p < 0.05, **p < 0.01, ***p < 0.001, n = 3/group. (D) Western blot showing SEMA6B expression levels in the AML12-SEMA6B^OE^ and AML12-SEMA6B^WT^ groups. (E) (a-b) Cytometric analysis of the cell cycle in the two groups at 24 h after LPS stimulation. (F) CCK-8 assay of cell proliferation in the two groups at 24 h, 48 h, and 72 h after LPS stimulation. (G) (a-b) Cytometric analysis of apoptosis in the two groups at 24 h after LPS stimulation. Mean ± SEM, *p < 0.05, **p < 0.01, n = 3/group. KD: knockdown; OE: overexpression; WT: wild-type; LPS: lipopolysaccharide; IL: interleukin; CCL: C-C motif chemokine ligand; ICAM: intercellular cell adhesion molecule.

We investigated the mechanisms underlying SEMA6B in macrophages and hepatocytes. SEMA6B overexpression (OE) and knockdown (KD) in mouse macrophages RAW264.7 were confirmed by Western blot analysis (Figure 4A). Analysis of proteomic data from RAW264.7-SEMA6B^OE^ cells revealed that differentially expressed proteins interacting with SEMA6B were enriched in inflammatory response processes, including responses to interleukin-4, epidermal growth factor, and tumor necrosis factor (Figure 4B, Table S2). To validate the role of SEMA6B, we induced an inflammatory model with LPS. RAW264.7-SEMA6B^OE^ cells exhibited significantly increased levels of proinflammatory cytokines (IL-6, IL-2, IL-1a, IL-1β, all p < 0.001), anti-inflammatory cytokines (IL-10, IL-12, all p < 0.001), and chemotactic factors (CCL2, ICAM1, all p < 0.001), while RAW264.7-SEMA6B^KD^ cells showed reduced cytokine production (Figure 4C (a-c)). SEMA6B overexpression in mouse hepatocytes AML12 was confirmed by Western blot (Figure 4D). SEMA6B overexpression significantly arrested the cell cycle of AML12 cells at the G0/G1 phase (Figure 4E (a-b)), resulting in reduced cell proliferation compared to AML12-SEMA6B^WT^ cells (Figure 4F). Apoptosis analysis revealed that SEMA6B overexpression induced significant apoptosis in AML12 cells (Figure 4G (a-b)). These findings suggest that SEMA6B may activate macrophage-mediated inflammatory responses and induce hepatocyte apoptosis, potentially contributing to liver failure progression.

Additionally, using SEMA6B siRNA in human macrophages (THP1-derived) and hepatocytes (HepaRG) replicated the observed effects similar to RAW264.7 and AML12 cells (Figure S6). These results provide additional validation for our findings.

### SEMA6B knockout alleviates liver failure

**Figure 5:**
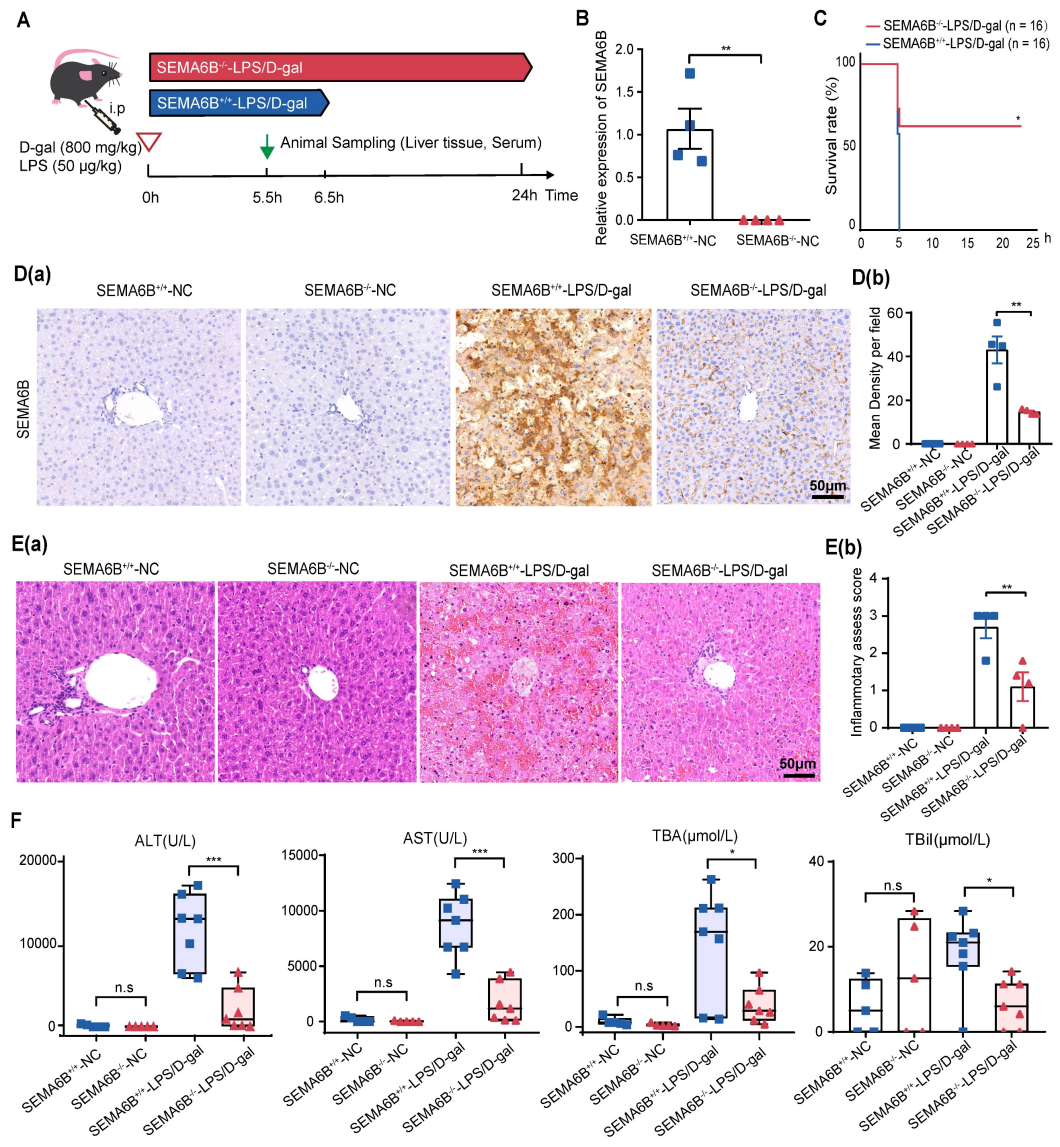
** Effects of SEMA6B deficiency in an LPS/D-gal-induced liver failure mouse model.** (A) Schematic design for modeling LPS/D-gal-induced liver failure. (B) Relative mRNA expression of SEMA6B in the two groups. Mean ± SEM, **p < 0.01, n = 4/group. (C) Survival analysis of mice in the two groups over 24h, *p < 0.05, n = 16/group. (D) (a-b) Immunohistochemical staining for SEMA6B in liver tissue from the four groups (bar = 50 μm). **p < 0.01, n = 4/group; five random fields analyzed per liver section. (E) (a) H&E staining of liver tissue from the four groups (bar = 50 μm); (b) inflammatory score of H&E staining in the four groups. Mean ± SEM, *p < 0.05, n = 4/group; five random fields analyzed per liver section. (F) Four typical biochemical indices of liver function detected in the four groups, mean ± SEM, *p < 0.05, ***p < 0.001, ns: not significant, n = 5-7/group. LPS: lipopolysaccharide; D-gal: D-galactose; ALT: alanine aminotransferase; AST: aspartate aminotransferase; TBA: total bile acid; TBil: total bilirubin.

To investigate the mechanisms of SEMA6B in liver failure, we employed conventional knockout (SEMA6B^-/-^) in embryonic C57BL/6 mice. SEMA6B knockout was validated through qRT-PCR and immunohistochemical staining (Figure 5B, Figure 5D). Survival analysis over a 24-hour post-LPS/D-gal injection period showed a significant rescue effect in the SEMA6B^-/-^-LPS/D-gal group, with 50% (8/16) surviving, compared to rapid mortality in the SEMA6B^+/+^-LPS/D-gal group (all deceased within 6.5 hours) (Figure 5C). Immunohistochemical staining demonstrated significantly lower SEMA6B expression in the SEMA6B^-/-^-LPS/D-gal group compared to the SEMA6B^+/+^-LPS/D-gal group (SEMA6B-positive area: 14.7 ± 0.623% vs. 42.0 ± 6.126%, p < 0.01) (Figure 5D (a-b)). H&E staining showed less hepatic necrosis, hemorrhage, and inflammatory cell infiltration in the SEMA6B^-/-^-LPS/D-gal group (inflammation score: 1.1 ± 0.300 vs. 2.7 ± 0.387, p < 0.05) (Figure 5E (a-b)). Biochemical analysis revealed significantly decreased levels of serum ALT (p < 0.001), AST (p < 0.001), TBA (p < 0.05), and TBil (p < 0.05) in the SEMA6B^-/-^-LPS/D-gal group compared to the SEMA6B^+/+^-LPS/D-gal group (Figure 5F). No significant differences were observed in these indexes between SEMA6B^+/+^-and SEMA6B^-/-^-normal groups. These findings suggest that SEMA6B knockout improves liver function, reduces hepatic necrosis, and prolongs survival in mice with liver failure.

### Effect on inflammatory response and hepatocyte apoptosis

**Figure 6:**
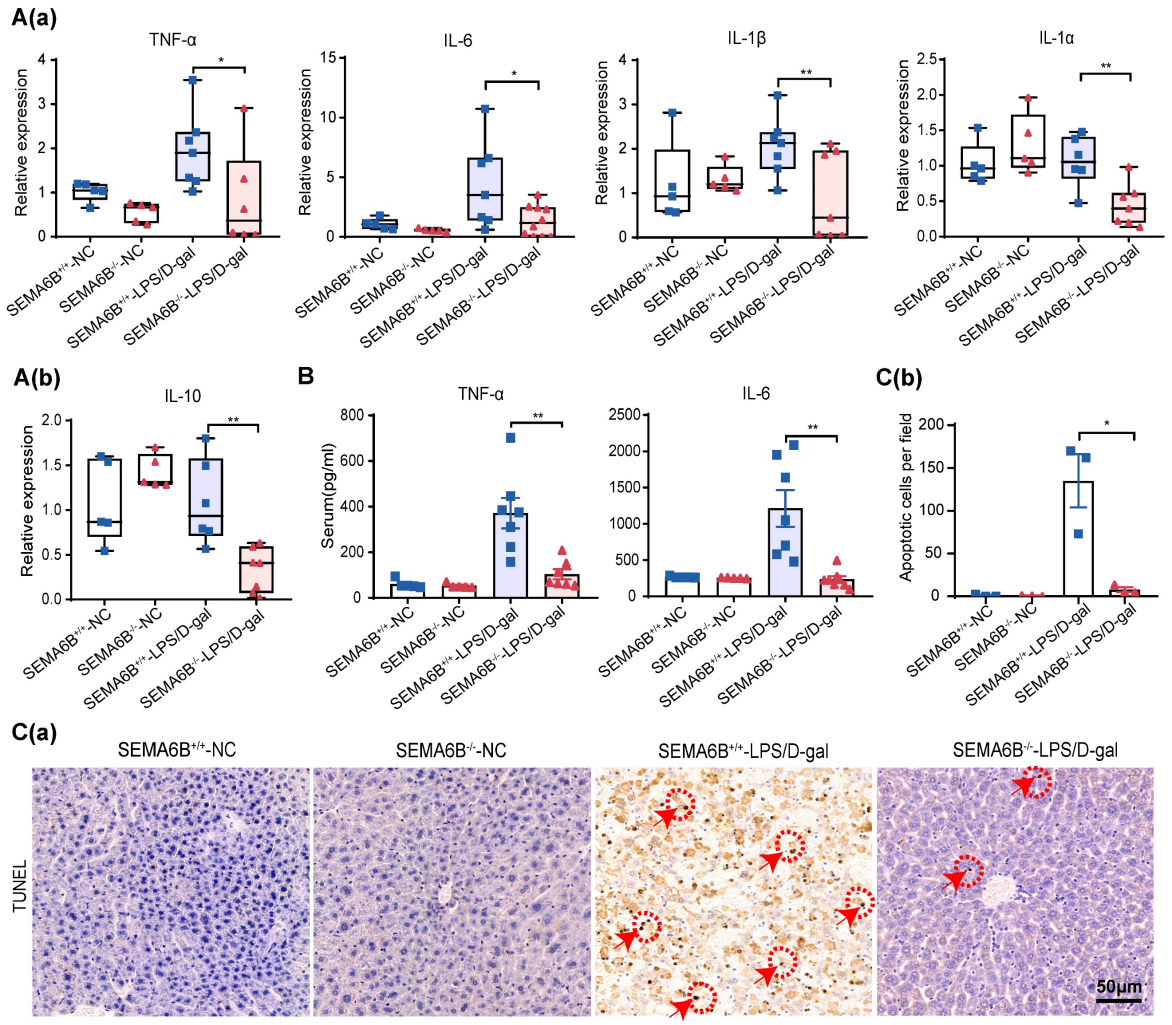
** Evidence of liver inflammation and hepatocyte apoptosis in SEMA6B^-/-^ mice with liver failure.** (A) Relative mRNA expression levels of five inflammatory markers in liver tissues collected from the four groups. Mean ± SEM, *p < 0.05, **p < 0.01, n = 5-7/group, ns: not significant. (B) ELISA detection of TNF-α and IL-6 levels in the serum of mice in the four groups; mean ± SEM, *p < 0.05, **p < 0.01, n = 5-7/group; ns: not significant. (C-a) TUNEL staining of liver tissue collected from the four groups (bar = 50 μm); (C-b) apoptotic cells per field of TUNEL staining in the four groups. Mean ± SEM, **p < 0.01, NA: not available, n = 4/group; five random fields were analyzed per liver section. The red arrow shows positive cells. LPS: lipopolysaccharide; D-gal: D-galactose; IL: interleukin.

qRT‒PCR analysis of liver tissues showed significant decreases in proinflammatory cytokines TNF-α, IL-6, IL-1β, and IL-1α (p < 0.05, p < 0.05, p < 0.01, and p < 0.01, respectively) and the anti-inflammatory cytokine IL-10 (p < 0.01) in SEMA6B^-/-^-LPS/D-gal group compared to SEMA6B^+/+^-LPS/D-gal group (Figure 6 (a-b)). Serum levels of TNF-α (p < 0.01) and IL-6 (p < 0.01) were also significantly reduced in SEMA6B^-/-^-LPS/D-gal group (Figure 6B). TUNEL staining analysis showed fewer apoptotic cells in SEMA6B^-/-^-LPS/D-gal group compared to SEMA6B^+/+^-LPS/D-gal group (p < 0.05) (Figure 6C (a-b)). These results further confirm that SEMA6B knockout attenuates the inflammatory response and reduces hepatocyte apoptosis in liver failure.

### SEMA6B knockout attenuates macrophage-mediated inflammatory responses and prevents hepatocyte apoptosis

**Figure 7:**
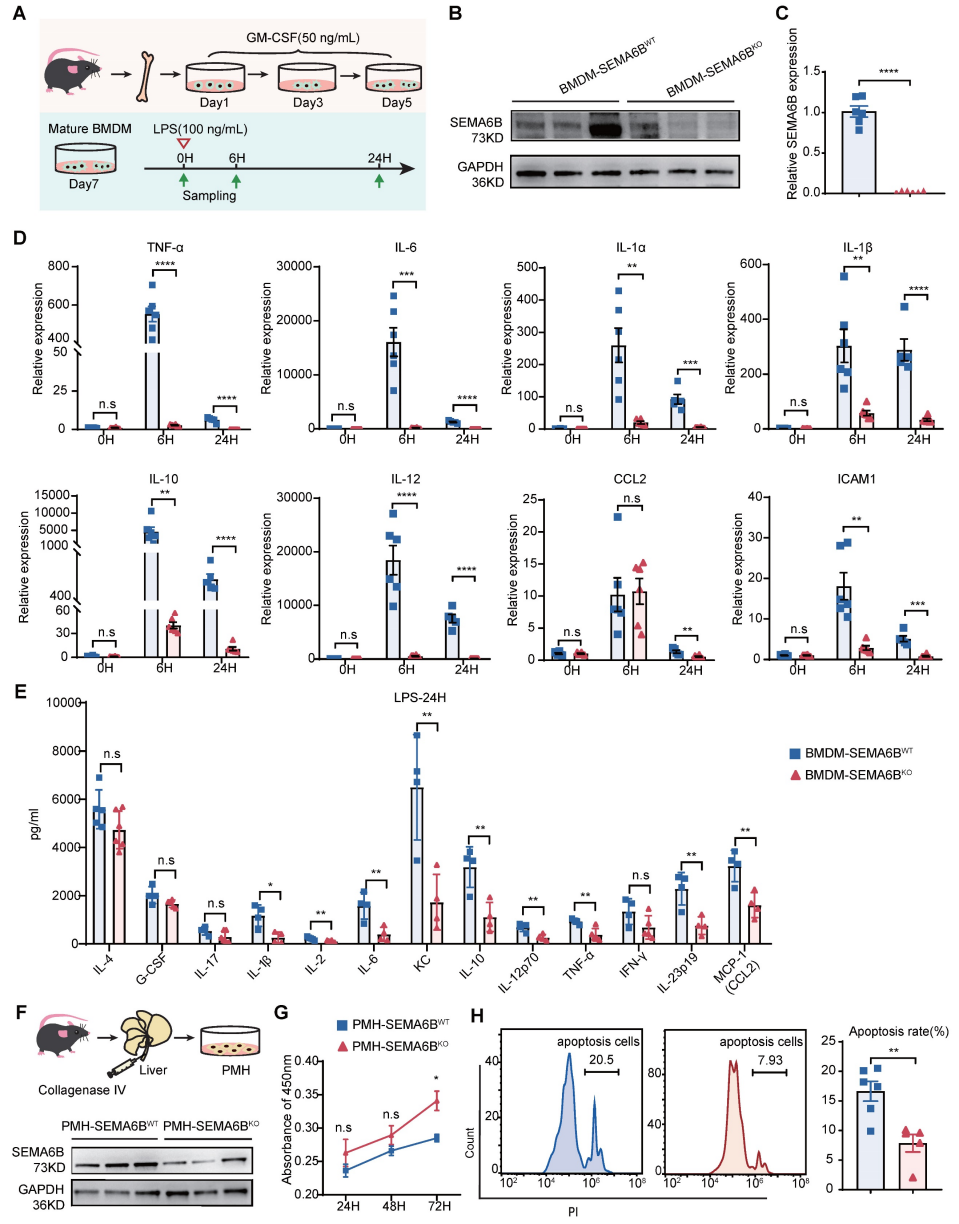
** Evidence for the functions of SEMA6B in primary macrophages and hepatocytes.** (A) Schematic design for isolation, culture and stimulation of BMDMs. (B) Western blot and (C) qRT-PCR showing the SEMA6B expression levels in the BMDM-SEMA6B^WT^ and BMDM-SEMA6B^KO^ groups. (D) The expression levels of the inflammatory cytokines BMDM-SEMA6B^WT^ and BMDM-SEMA6B^KO^ groups at 0 h, 6 h, and 24 h after LPS stimulation. (E) The levels of the inflammatory cytokines in the supernatants of BMDM-SEMA6B^WT^ and BMDM-SEMA6B^KO^ groups at 24 h after LPS stimulation. ns: not significant, *p < 0.05, **p < 0.01, ***p < 0.001, ****p < 0.0001, n = 4-6/group. (F) Schematic design for isolation of PMHs. Western blotting showing the SEMA6B expression levels in the PMH-SEMA6B^WT^ and PMH-SEMA6B^KO^ groups (G) CCK-8 detection of cell proliferation in the two groups at 24 h, 48 h, and 72 h after LPS stimulation. (H) Cytometric analysis of apoptosis in the two groups at 24 h after LPS stimulation. Mean ± SEM, ns: not significant, *p < 0.05, **p < 0.01, n=4/group. BMDMs: bone marrow-derived macrophages; PMHs: primary mouse hepatocytes; KD: knockdown; OE: overexpression; WT: wild-type; LPS: lipopolysaccharide; IL: interleukin; CCL: C-C motif chemokine ligand; ICAM: intercellular cell adhesion molecule.

To elucidate the mechanisms underlying SEMA6B's role, we investigated its impact on primary mouse macrophages and hepatocytes. Bone marrow-derived macrophages (BMDMs) were isolated, cultured, and stimulated, as depicted in Figure 7A. SEMA6B knockout in BMDMs was confirmed through Western blot and qRT-PCR analyses (Figure 7B-C). Both mRNA and protein levels of inflammatory cytokines were significantly reduced in BMDM-SEMA6B^KO^ cells, indicating that SEMA6B knockout attenuates macrophage-mediated inflammatory responses (Figure 7D-E). Primary mouse hepatocytes (PMHs) were prepared and cultured, as illustrated in Figure 7F. Western blot analysis confirmed SEMA6B knockout in PMHs (Figure 7F). SEMA6B knockout in PMHs significantly enhanced cell proliferation and suppressed apoptosis (Figure 7G-H). These findings suggest that silencing SEMA6B may protect against hepatocyte apoptosis.

### Transcriptomic characteristics of SEMA6B knockout mice with liver failure

**Figure 8:**
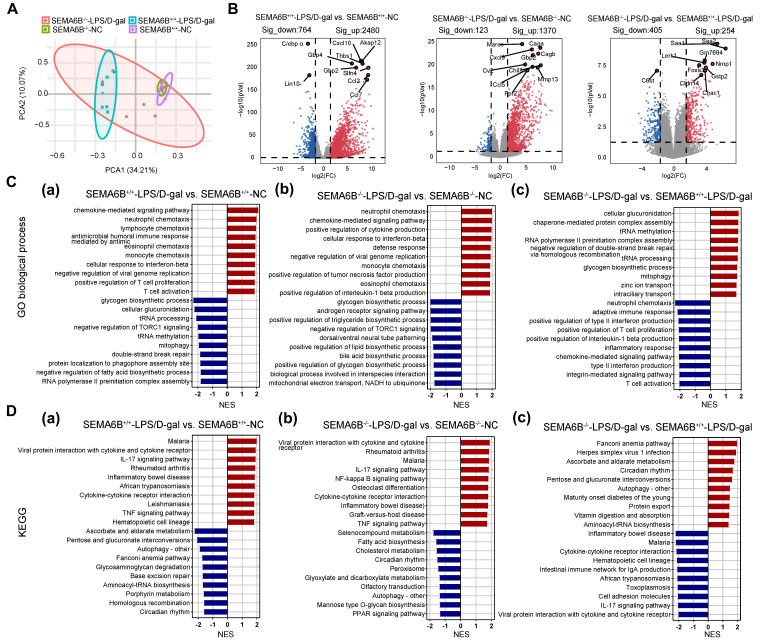
** Transcriptomic characteristics of SEMA6B knockout mice with LPS/D-gal-induced liver failure.** (A) Principal component analysis of liver tissues from the four groups (n = 5-7/group). (B) The top 10 significant DEGs from the pairwise comparison of SEMA6B^-/-^-LPS/D-gal versus SEMA6B^-/-^-NC, SEMA6B^+/+^-ALF versus SEMA6B^+/+^-NC and SEMA6B^-/-^-LPS/D-gal versus SEMA6B^+/+^-LPS/D-gal are shown in a volcano plot. Significantly differentially expressed genes (|log2Fold Change| > 3, adjusted p value < 0.05) are shown in red (upregulated) and blue (downregulated). (C) Identification of the top 20 significantly upregulated (red) and downregulated (blue) biological processes and (D) KEGG pathways in three pairwise comparisons between the two groups. Enrichment analysis was performed using gene set enrichment analysis, with red (up) and blue (down) indicating the normalized enrichment score of each gene set. LPS: lipopolysaccharide; D-gal: D-galactose; GO, Gene Ontology; KEGG, Kyoto Encyclopedia of Genes and Genomes; NES, normalized enrichment score.

PCA based on liver tissues transcriptome data revealed the distinct separation of the SEMA6B^-/-^-LPS/D-gal group from the SEMA6B^+/+^-LPS/D-gal group and the two NC groups (Figure 8A). Pairwise differential expression analysis identified the 2480/764 (significantly up/down) DEGs in the SEMA6B^+/+^-LPS/D-gal group compared to SEMA6B^+/+^-NC group, and the 1370/123 (significantly up/down) DEGs in SEMA6B^-/-^-LPS/D-gal group compared to SEMA6B^-/-^-NC group (Figure 8B). Comparison between the SEMA6B^-/-^-LPS/D-gal and SEMA6B^+/+^-LPS/D-gal groups showed 405/254 (significant up/down) DEGs (Figure 8B), indicating SEMA6B knockout significantly altered the gene expression profile in mice with liver failure.

### Effects of inflammation processes in the SEMA6B knockout model

To further elucidate the pathophysiological changes associated with SEMA6B knockout in liver failure, pre-ranked gene set enrichment analysis identified the top 20 significantly regulated biological processes. Pairwise Gene Ontology (GO) functional analysis and Kyoto Encyclopedia of Genes and Genomes (KEGG) pathway analysis were conducted to explore correlated pathways.

Compared to SEMA6B^+/+^-NC mice, SEMA6B^+/+^-LPS/D-gal mice exhibited significant upregulation of inflammatory biological processes, including chemotactic factor-mediated pathways, responses to type II interferons, immune cell chemotaxis, and activation of T-cell proliferation (Figure 8C(a)). KEGG analysis also revealed upregulation of inflammatory pathways such as viral protein interactions, cytokine/cytokine receptor interactions, the IL-17 signaling pathway, the TNF signaling pathway, and cytokine-cytokine receptor interaction (Figure 8D(a)). In contrast, SEMA6B^-/-^-LPS/D-gal mice demonstrated increased inflammatory processes such as neutrophil and monocyte chemotaxis, positive regulation of cytokine production (tumor necrosis factor, IL-1β), and defense response compared to SEMA6B^-/-^-NC mice (Figure 8C(b)). KEGG analysis further confirmed the upregulation of inflammatory pathways in SEMA6B^-/-^-LPS/D-gal mice, including viral protein interactions, cytokine/cytokine receptor interactions, the IL-17 signaling pathway, the TNF signaling pathway, and the NF-κB signaling pathway (Figure 8D(b)). These results highlight significant upregulation of inflammation-related pathways in both wild-type and SEMA6B knockout mice with liver failure.

However, functional enrichment analysis in the two groups of mice with liver failure showed that SEMA6B knockout significantly downregulated inflammation-related biological processes, including positive regulation of type II interferon production, positive regulation of IL-1β production, pathways mediated by chemotactic factors, integrin-mediated pathways, and adaptive immune responses such as immune cell chemotaxis, proliferation, and activation of T cells (Figure 8C(c)). KEGG analysis further confirmed these results by demonstrating decreased expression of inflammatory pathways, such as viral protein interactions, cytokine/cytokine receptor interactions, the IL-17 signaling pathway, the TNF signaling pathway, and cytokine‒cytokine receptor interactions (Figure 8D(c)). These findings indicate that SEMA6B knockout can downregulate inflammation in mice with liver failure, thereby potentially alleviating inflammation-mediated liver failure.

### Treatment with synthetic SEMA6B siRNA in mice with liver failure

**Figure 9:**
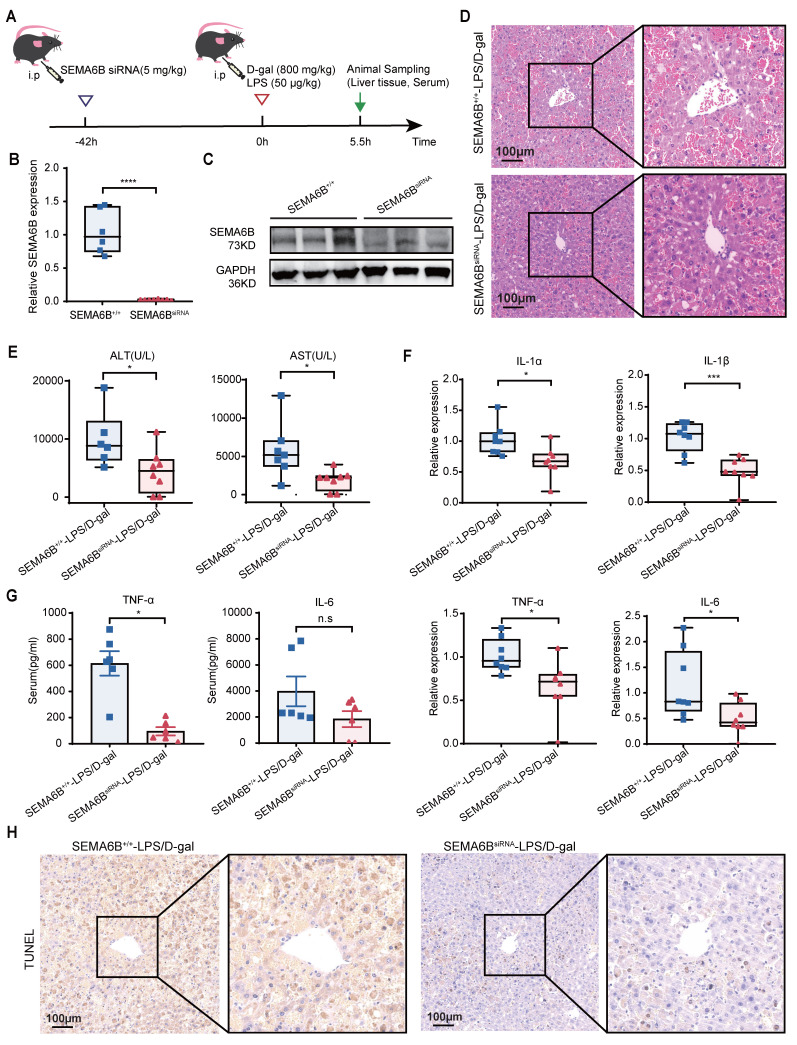
** Effects of treatment of SEMA6B siRNA in liver failure mouse models.** (A) Schematic design for administrating SEMA6B siRNA in mouse models of liver failure. (B) qRT-PCR and (C) Western blot showing the SEMA6B expression levels in the SEMA6B^+/+^ and SEMA6B^siRNA^ groups. (D) H&E staining of liver tissue from the two groups (bar = 100 μm). (E) Two classical biochemical indexes of liver function detected in the two groups, mean ± SEM, *p < 0.05, n = 6-8/group. (F) Relative mRNA expression levels of four inflammatory markers in liver tissues collected from the two groups. Mean ± SEM, *p < 0.05, ***p < 0.001, n = 8/group. (G) ELISA detection of the TNF-α and IL-6 levels in the serum of mice in the two groups; mean ± SEM, *p < 0.05, ns: not significant, n = 6/group. (H) TUNEL staining of liver tissue collected from the two groups (bar = 100 μm), n = 6-8/group.

To explore the clinical potential of SEMA6B, we administered synthetic SEMA6B siRNA in a mouse model of liver failure, as depicted in Figure 9A. The SEMA6B knockdown in mice was confirmed through qRT-PCR and Western blot analyses (Figure 9B-C). Injection of synthetic SEMA6B siRNA significantly reduced massive hepatic necrosis and hemorrhage, and facilitated liver structure restoration in mice with liver failure (Figure 9D). Furthermore, there was a significant improvement in liver function, as evidenced by decreased levels of ALT and AST (p < 0.05) (Figure 9E). Administration of synthetic SEMA6B siRNA led to a reduction in inflammatory cytokine levels in mice with liver failure, assessed by qRT-PCR and ELISA (Figure 9F-G). Moreover, hepatocyte apoptosis was mitigated following treatment with synthetic SEMA6B siRNA (Figure 9H). These results underscore the therapeutic potential of synthetic SEMA6B siRNA delivery in vivo for alleviating inflammation and hepatocyte apoptosis, thereby proposing a novel therapeutic strategy for liver failure.

## Discussion

Acute-on-chronic liver failure (ACLF) poses a significant clinical challenge due to its complex pathophysiology and high short-term mortality rates^1^-^3^. Effective early diagnosis and accurate prognostic strategies are urgently needed to improve patient outcomes^15^. Current diagnostic and prognostic approaches for ACLF rely on complex multiorgan failure scores with clinical and laboratory indicators. However, there remains a critical need for simple, easily accessible biomarkers to enhance early detection and prediction of ACLF^16^-^21^.

In this study, SEMA6B emerged as a prominent gene through multigroup comparisons in high-throughput transcriptome analysis of a prospective multicenter cohort. It exhibited the most reliable diagnostic performance for ACLF progression. Previous research has also highlighted elevated SEMA6B levels in HBV-ACLF patients, showing its potential to distinguish ACLF from chronic liver disease with high sensitivity and specificity^12^. Our validation in an external cohort using qRT‒PCR on peripheral blood mononuclear cells and immunofluorescence in liver tissues confirmed the high expression of SEMA6B, particularly in ACLF non-survivors. A recent study further corroborated these findings by demonstrating high SEMA6B expression in an ACLF rat model^6^ , thereby reinforcing the reliability of these conclusions. Nonetheless, additional validation in larger multicenter cohorts is necessary to more accurately evaluate the clinical utility of SEMA6B as a biomarker.

The correlation of SEMA6B levels with disease severity can facilitate the typing and staging of the disease. ACLF mortality increased with the ACLF grade. Furthermore, INR and coagulation failure have been used as core prognostic markers in CLIF-C ACLFs, MELD-Na, MELD, and COSSH-ACLF Ⅱs^3^, ^22^-^24^. In this study, we observed that SEMA6B levels significantly increased with ACLF grades, and were positively correlated with INR and ALT indicators. We highlighted the SEMA6B-correlated genes involved in pathways involved in the progression of ACLF non-survivors, including inflammation, complement and coagulation cascades, oxidative phosphorylation, and apoptosis. Our previous study further demonstrated that the SEMA6B gene plays a role in immune inflammatory responses, complement activation, apoptosis, and reactive oxygen species metabolism^6^. These findings suggest that SEMA6B levels are linked to inflammation, apoptosis, and metabolic processes driving ACLF development and progression. However, further cell and animal studies are needed to elucidate the precise molecular mechanisms by which SEMA6B influences inflammation and apoptosis in ACLF progression.

Understanding the cell types that express SEMA6B in ACLF is essential for elucidating its underlying mechanisms. Publicly available databases indicated that SEMA6B was expressed in myeloid immune cells within healthy PBMCs, including neutrophils, classical monocytes, and myeloid dendritic cells, as well as in hepatocytes, macrophages, and endothelial cells within healthy liver tissue. Previous studies have shown that SEMA6B was a marker for macrophages and circulating monocytes and was expressed in physiological endothelial cells^10^, ^11^, ^25^. Although SEMA6B expression is significantly related to the stage of liver cirrhosis in HCV-infected patients, its relevance to hepatocytes had not been previously reported^9^. In our study, we found high SEMA6B expression in ALB-positive hepatocytes and CD86-positive macrophages in the liver tissue of HBV-ACLF patients. However, SEMA6B expression was not detected in endothelial cells from these patients, which may be attributed to the rapid dedifferentiation of endothelial cells during acute and chronic liver injury^26^, resulting in changes to their functional properties. Consequently, it is believed that macrophages and hepatocytes play significant roles in the involvement of SEMA6B in the development and progression of ACLF.

Understanding the molecular mechanisms underlying the involvement of SEMA6B in ACLF progression is crucial for developing novel treatment strategies. Systemic inflammation is a key factor driving ACLF development and progression^27^. During the chronic stage, intense systemic inflammation primarily results from infection and extensive hepatocyte damage, as evidenced by elevated levels of pro-(IL-6, TNF-α, IL-1α)/anti-(IL-10) inflammatory cytokines^27^-^29^. In this study, genes significantly associated with high levels of SEMA6B in RAW264.7 cells were enriched in inflammatory biological processes. SEMA6B overexpression significantly increased the levels of inflammatory cytokines, including IL-6, IL-10, IL-1α, IL-1β, IL-2, IL-12, the chemokine CCL2, and the adhesion molecule ICAM-1, in RAW264.7 cells. Conversely, knockdown of SEMA6B in THP1 and RAW264.7 cells, as well as in primary SEMA6B knockout macrophages, resulted in a significant downregulation of these cytokines. Additionally, SEMA6B overexpression in AML12 cells induced hepatocyte apoptosis, while silencing SEMA6B reduced apoptosis in SEMA6B knockdown HepaRG cells and primary SEMA6B knockout hepatocytes. These results suggest that SEMA6B can promote both inflammation and hepatocyte apoptosis. Recent studies have demonstrated that intense systemic inflammation is sustained by circulating pathogen-associated molecular patterns (PAMPs) and damage-associated molecular patterns (DAMPs)^30^-^32^. These patterns interact with their pattern recognition receptors (PRRs) to drive the expression of inflammatory cytokines, such as IL-6, TNF-α, and IL-1β, leading to systemic inflammation and cell death^33^. To model this, we used LPS and D-gal as mimics of PAMPs and DAMPs to develop a liver failure mouse model. SEMA6B knockout alleviated LPS/D-gal-induced liver failure, improved liver function, reduced inflammatory responses, and decreased hepatocyte apoptosis. These effects were also confirmed by the treatment of liver failure mice with synthetic SEMA6B siRNA. Sequencing data from liver tissues indicated that SEMA6B knockout significantly improved the transcriptome profiles of mice with liver failure. Functional analysis revealed a significant downregulation of inflammation-related pathways in the liver tissues of SEMA6B knockout mice. These findings align with previous research showing that semaphorins are involved in crucial physiological and pathological immune responses, including immune cell activation, differentiation, and trafficking^34^. SEMA6B has also been linked to inflammatory responses and apoptosis^35^-^37^. Targeting SEMA6B could mitigate macrophage-mediated systemic inflammatory responses and hepatocyte apoptosis induced by PAMPs and DAMPs, providing a novel therapeutic target.

However, our study also has certain limitations. Firstly, due to the lack of suitable HBV-ACLF mouse models, we used the LPS/D-gal-induced acute liver failure (ALF) mouse model for mechanistic studies. Although ALF and ACLF exhibit some clinical symptoms, significant differences between these disease entities cannot be ruled out. Second, Secondly, we used SEMA6B embryonic knockout mice to study the mechanisms of SEMA6B in liver failure due to the limited availability of SEMA6B macrophage- and hepatocyte-specific knockout mice. While this study cannot verify the specific molecular mechanisms involving SEMA6B in ACLF, it utilized clinical samples, screened SEMA6B via high-throughput transcriptome sequencing, and validated the findings in an external cohort. Additionally, mechanisms of SEMA6B were validated in vitro using hepatocytes and macrophages from mouse and human cell lines, and primary cells, as well as in vivo with SEMA6B knockout mice and synthetic SEMA6B siRNA treatment. Therefore, the conclusions drawn from this study remain robust. Finally, our study was limited to ACLF patients with a single etiology of HBV, restricting the applicability of this biomarker. Future studies should validate these findings in a cohort of ACLF patients with diverse etiologies.

## Conclusions

In conclusion, SEMA6B is highly expressed in HBV-ACLF patients and has accurate diagnostic value for HBV-ACLF. SEMA6B exacerbates HBV-ACLF progression by inducing macrophage-mediated systemic inflammatory responses and hepatocyte apoptosis. Therefore, targeting SEMA6B may be a promising early treatment therapeutic strategy to prevent HBV-ACLF progression.

## Supplementary Material

Supplementary methods, figures and tables.

## Figures and Tables

**Table 1:** Clinical characteristics of the patients

Characteristics	HBV-ACLF (n = 142)	LC (n = 60)	CHB (n = 60)	NC (n = 59)
Age (years)	45.0 (36.0, 57.0)	46.0 (40.3, 53.8)	42.0 (37.0, 50.5) *	47.0 (33.0, 53.0)
Male (%)	85.2% (121)	73.3% (44)	85% (51)	55.9% (33) ***
**HBV DNA level (IU/mL)**				
≤ 2×10^2^	4.2% (6)	88.3% (53) ***	88.3% (53) ***	NA
2×10^2^ - 2×10^6^	64.8% (92)	11.7% (7) ***	6.7% (4) ***	NA
> 2×10^6^	31.0% (44)	NA	5% (3) ***	NA
**Laboratory data**				
Alanine aminotransferase (U/L)	254.5 (90.0, 564.8)	25.0 (18.0, 33.8) ***	24.5 (19.3, 32.8) ***	19.0 (14.0, 25.0) ***
Aspartate aminotransferase (U/L)	143.0 (84.0, 284.0)	24.0 (19.3, 30.0) ***	24.0 (20.0, 27.8) ***	20.0 (18.0, 25.0) ***
Albumin (g/L)	31.6 (28.2, 33.8)	47.5 (45.6, 49.0) ***	49.0 (46.7, 51.3) ***	47.7 (46.1, 49.1) ***
Total bilirubin (µmol/L)	344.5 (261.0, 432.3)	13.2 (11.0, 16.8) ***	12.3 (8.0, 16.0) ***	11.0 (8.6, 14.0) ***
Alkaline phosphatase (U/L)	139.5 (112.0, 169.0)	70.0 (57.3, 90.0) ***	63.0 (48.0, 79.3) ***	67.5 (55.0, 84.0) ***
γ-glutamyl transpeptidase (U/L)	78.5 (53.8, 118.5)	23.5 (17.5, 36.5) ***	24.5 (15.3, 35.0) ***	19.0 (12.0, 27.0) ***
Creatinine (μmol/L)	65.5 (56.75, 79.25)	72.0 (62.8, 81.3)	77.0 (65.0, 85.0) ***	70.0 (57.0, 81.0)
Sodium (mmol/L)	137.0 (135.0, 139.0)	141.0 (140, 143.0) ***	141.0 (140.0, 143.0) ***	141.0 (140.0, 143.0.0) ***
White blood cell count (10^9^/L)	7.6 (6.0, 9.6)	5.4 (4.1, 6.5) ***	5.6 (4.9, 6.4) ***	6.1 (5.4, 6.9) ***
Hemoglobin (g/L)	124.0 (112.5, 136.0)	153.0 (138.5, 159.0) ***	154.0 (143.0, 164.0) ***	148.0 (136.0, 160.0) ***
Hematocrit (%)	35.6 (31.7, 39.3)	45.0 (41.0, 47.2) ***	45.0 (42.1, 47.1) ***	44.0 (40.7, 48.3) ***
Platelet count (10^9^/L)	108.0 (71.5, 141.0)	162.0 (117.3, 211.0) ***	178.0 (142.0, 218.0) ***	219.0 (187.0, 261.0) ***
INR	2.3 (1.8, 2.8)	1.0 (1.0, 1.1) ***	1.0 (0.9, 1.0) ***	NA
Alpha fetoprotein (μg/L)	88.8 (29.6, 223.2)	2.2 (1.8, 2.8) ***	2.5 (1.9, 3.5) ***	2.7 (1.9, 4.0) ***
**Organ failure (%)**				
Liver	95.1% (135)	0***	0***	0***
Coagulation	38.7% (55)	0***	0***	0***
Kidneys	3.5% (5)	0	0	0
Brain	5.6% (8)	0	0	0
Lungs	0.7% (1)	0	0	0
Circulation	1.4% (2)	0	0	0
**Severity score**				
COSSH-ACLF ⅠⅠs	6.5 (5.8, 7.4)	NA	NA	NA
COSSH-ACLFs	6.0 (5.4, 6.7)	NA	NA	NA
CLIF-C ACLFs	40.6 (36.8, 45.3)	NA	NA	NA
MELD-Nas	26.8 (22.6, 32.5)	NA	NA	NA
MELDs	25.9 (21.7, 32.2)	NA	NA	NA
**Transplant-free mortality rate (number of deceased patients)**				
28-day	30.4% (38)	0***	0***	NA
90-day	42.9% (51)	0***	0***	NA

Notes: Data are presented as medians (p25, p75) or percentages (numbers of patients). Eleven patients with ACLF underwent liver transplantation and were considered lost to follow-up in the mortality rate calculation. Six patients with ACLF were lost to follow-up at 28 days, and twelve patients were lost to follow-up at 90 days. *p < 0.05, **p < 0.01 and ***p* <* 0.001 for comparisons between the HBV-ACLF group and the LC/CHB/NC group. ACLF, acute-on-chronic liver failure; LC, liver cirrhosis; CHB, chronic hepatitis B; NC, normal control. COSSH-ACLF ⅠⅠs, COSSH-ACLF ⅠⅠ score; COSSH-ACLFs, COSSH-ACLF score; CLIF-C ACLFs, CLIF Consortium ACLF score; MELD-Nas, Model for End-stage Liver Disease-serum Na score; MELDs, Model for End-stage Liver Disease score.

## Abbreviations

HBV-ACLF: hepatitis B virus-related acute-on-chronic liver failure
LC: liver cirrhosis
CHB: chronic hepatitis B
NC: normal control
COSSH: Chinese group on the study of severe hepatitis B
SEMA6B: semaphorin-6B
AUROC: area under the ROC
INR: international normalized ratio
ALT: alanine transaminase
TNF: tumor necrosis factor
IL: interleukin
PCA: principal component analysis
DEGs: differentially expressed genes
RT‒qPCR: reverse transcription quantitative real-time PCR
GSEA: gene set enrichment analysis
PBMCs: peripheral blood mononuclear cells
ALB: albumin
CLD: chronic liver disease
CCL: chemokine c-c motif ligand
ICAM1: intercellular adhesion molecule
LPS: lipopolysaccharide
D-gal: D-galactose
OE: overexpression
KD: knockdown
KO: knockout
GO: Gene Ontology
KEGG: Kyoto Encyclopedia of Genes and Genomes
