# Supplementary material for: SEMA6B induces macrophage-mediated inflammation and hepatocyte apoptosis in hepatitis B virus-related acute-on-chronic liver failure
Source: Theranostics. 2024 Aug 19;14(13):5200–18. doi: 10.7150/thno.97007 (PMC11388073; doi:10.7150/thno.97007)
Supplement: Supplementary file 1 — Supplementary methods, figures and tables. [file thnov14p5200s1.pdf]

SEMA6B induces macrophage-mediated inflammation and hepatocyte apoptosis in hepatitis B virus-related acute-on-chronic liver failure

Table of Contents

SUPPLEMENTARY METHODS..2

SUPPLEMENTARY FIGURES..5

SUPPLEMENTARY TABLES..12

SUPPLEMENTARY METHODS

Real-Time quantitative PCR (qRT-PCR)

Total RNA was extracted from human peripheral blood mononuclear cells (PBMCs) and mouse liver tissues using TRIzol reagent, following the manufacturer's instructions. The quality and concentration of the RNA samples were measured using a NanoDrop 2000 spectrophotometer (Thermo Fisher Scientific). cDNA was synthesized from the extracted RNA using the HiScript II Q RT SuperMix for qPCR (+gDNA) (Vazyme). Quantitative RT-PCR was then performed using the ChamQ Universal SYBR Master Mix (Vazyme), according to the manufacturer's protocol. The qRT-PCR was conducted using the ProFlex Base (Thermo Fisher Scientific) and the 7500 Real-Time PCR System (Thermo Fisher Scientific). Gene expression levels were normalized to the housekeeping gene β-actin, with target primer sequences listed in Table S1. Relative expression levels were calculated using the $2^{-\Delta\Delta Ct}$ method, with samples from the NC group or non-survivors serving as the reference for normalization.

Western blotting

Treated cell lines were lysed in RIPA buffer (Beyotime) containing phosphatase inhibitors (Roche) and protease inhibitors (Roche). Protein concentrations were determined using a BCA protein assay kit (Beyotime) and measured with a Microplate Reader (Thermo Fisher Scientific). Proteins were separated on ExpressPlus PAGE gels (Genscript) and transferred onto nitrocellulose membranes according to the manufacturer's instructions. Membranes were blocked with QuickBlock (Beyotime) and then incubated overnight at 4°C with primary antibodies against SEMA6B (Santa Cruz Biotechnology, 1:500 dilution) and β-actin (Abcam, 1:1000 dilution). After five washes with Tris-Buffered Saline containing Tween-20 (TBST), membranes were

incubated for 1 hour at room temperature with HRP-conjugated secondary antibodies (Abcam, 1:1000 dilution). The membranes were then washed again with TBST. Protein detection was carried out using Super ECL Detection Reagent (Yeasen), and the immunoblots were visualized using an imaging system. The specific antibodies used are listed in Table S3.

Immunohistochemistry (IHC) and immunofluorescence (IF)

To observe SEMA6B expression in liver tissues from patients with HBV-ACLF, LC, CHB, NC, as well as in mouse liver tissues, immunohistochemistry staining was conducted. Tissue slices (4 μm) were cut from paraffin-embedded liver sections, then deparaffinized and rehydrated using xylene and a gradient of ethanol solutions. Endogenous peroxidase activity was blocked with periodic acid (0.05% w/v in deionized water) for 10 minutes at room temperature. Antigen retrieval was performed using a microwave with Tris-EDTA buffer (pH 9) or Citrate buffer (pH 6), depending on the application. Following antigen retrieval, the slices were blocked with 5% (w/v) bovine serum albumin (BSA) in PBS, gently shaken for 1 hour at room temperature, and then incubated overnight at 4°C with a primary antibody against human or mouse SEMA6B (Santa Cruz Biotechnology, 1:50 dilution). After five washes with PBS containing 0.05% (v/v) Tween-100, the sections were incubated for 1 hour at 37°C with an HRP-conjugated secondary antibody (Abcam, 1:1000 dilution) and washed again with the same buffer. Finally, the sections were stained using an HRP kit and counterstained with hematoxylin. Immunostaining was examined under a microscope.

The percentage of SEMA6B-positive cells in liver tissue sections was assessed using Image-Pro Plus software. Five random fields per section were selected, and three liver

tissue sections were analyzed per group. Liver tissue samples were obtained from three subjects in each of the HBV-ACLF, LC, CHB, and NC groups.

H&E staining

Slices (4 μm) were cut from paraffin-embedded mouse liver sections for H&E staining. Each liver tissue section was heat-fixed at 60°C for 1 hour and then stained with hematoxylin and eosin (H&E). The inflammatory score, including portal and lobular scores, was graded as follows: 0, no inflammation; 1, mild lobular inflammation (< 10% of liver parenchyma) / portal inflammation (< 1/3 of portal tracts); 2, moderate lobular inflammation (10-50% of liver parenchyma) / portal inflammation (approximately 50% of portal tracts); and 3, severe lobular inflammation (> 50% of liver parenchyma) / portal inflammation (> 2/3 of portal tracts). Liver tissue samples were harvested from four mice in each of the wild-type and SEMA6B knockout normal control groups, as well as the wild-type and SEMA6B knockout liver failure groups.

SUPPLEMENTARY FIGURES

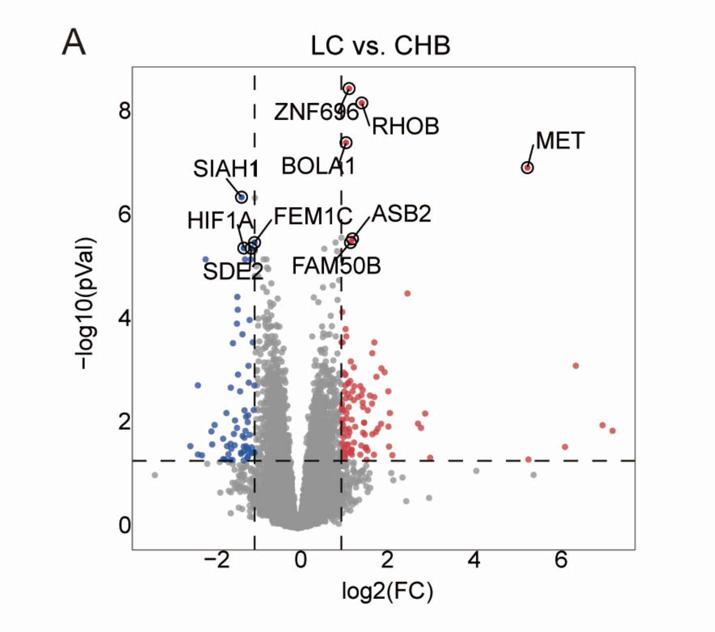

Figure S1. Volcano plot depicting the top 10 significant differentially expressed genes (DEGs) from the pairwise comparison of LC versus CHB. Genes with significant differential expression (|log2-fold change| > 1; adjusted p-value < 0.05) are highlighted in red (upregulated) and blue (downregulated).

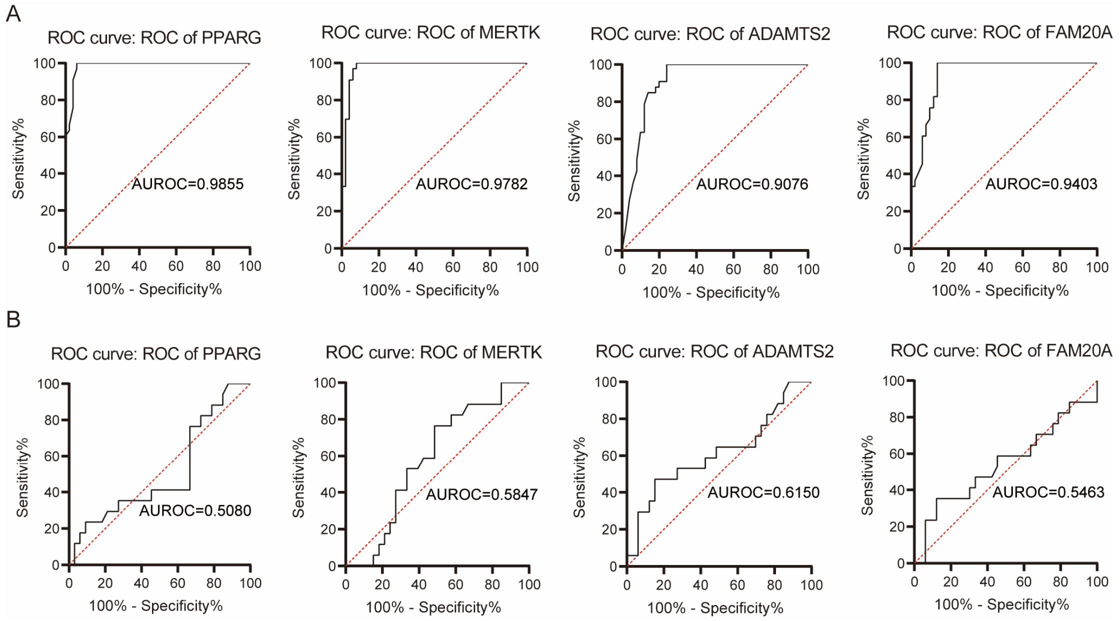

Figure S2. ROC analysis of the predictive capability of PPARG, MERTK, ADAMTS2, and FAM20A

(A) ROC curves for PPARG, MERTK, ADAMTS2, and FAM20A levels in distinguishing patients with HBV-ACLF from those with LC, CHB, and normal controls. (B) ROC curves for PPARG, MERTK, ADAMTS2 and FAM20A levels in distinguishing HBV-ACLF survivors from non-survivors.

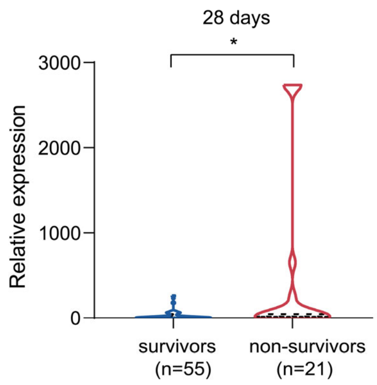

Figure S3. SEMA6B levels in HBV-ACLF survivors and non-survivors in the external validation cohort.

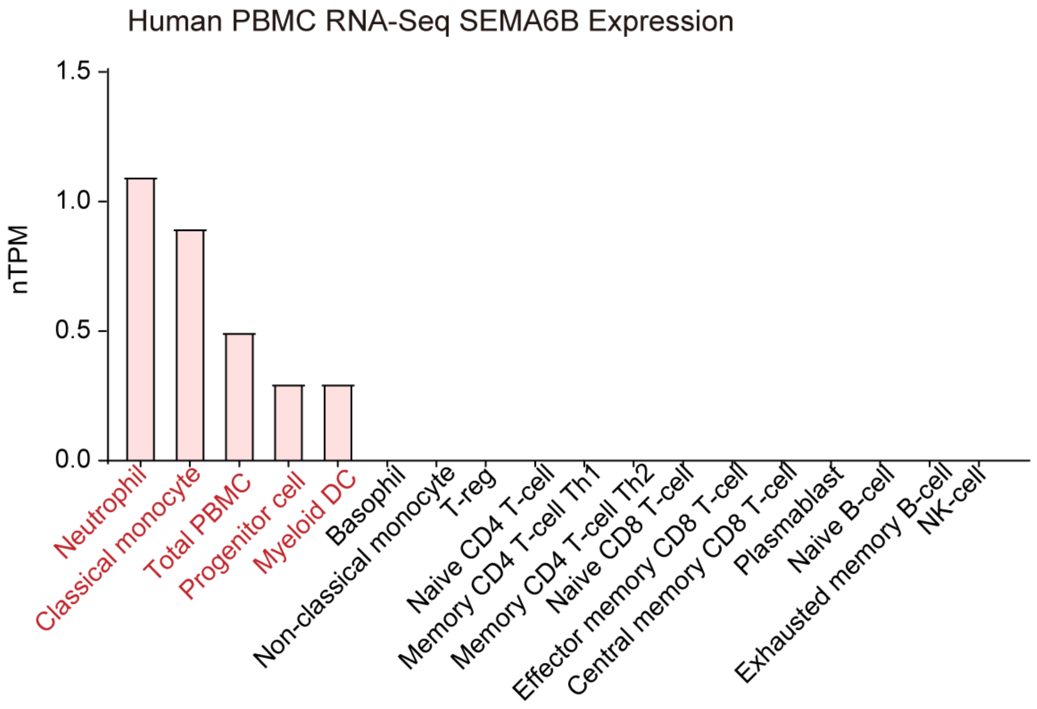

Figure S4 Cell types expressing SEMA6B in RNA-seq data from healthy peripheral blood mononuclear cells (PBMCs).

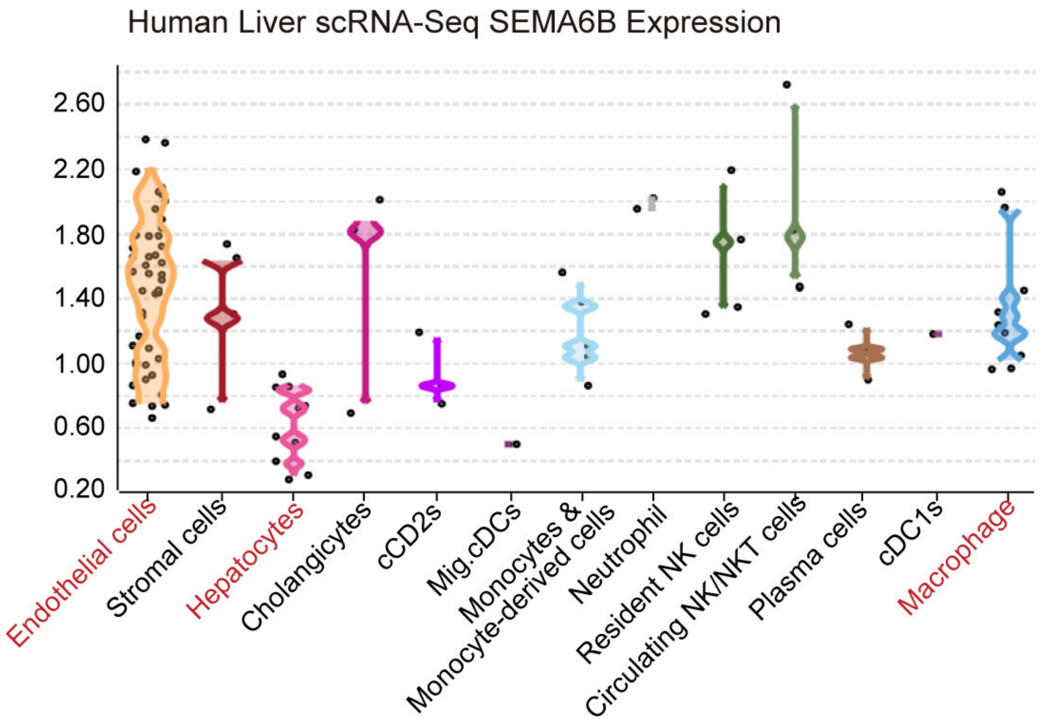

Figure S5 Cell types expressing SEMA6B in single-cell RNA-seq data from healthy liver tissues.

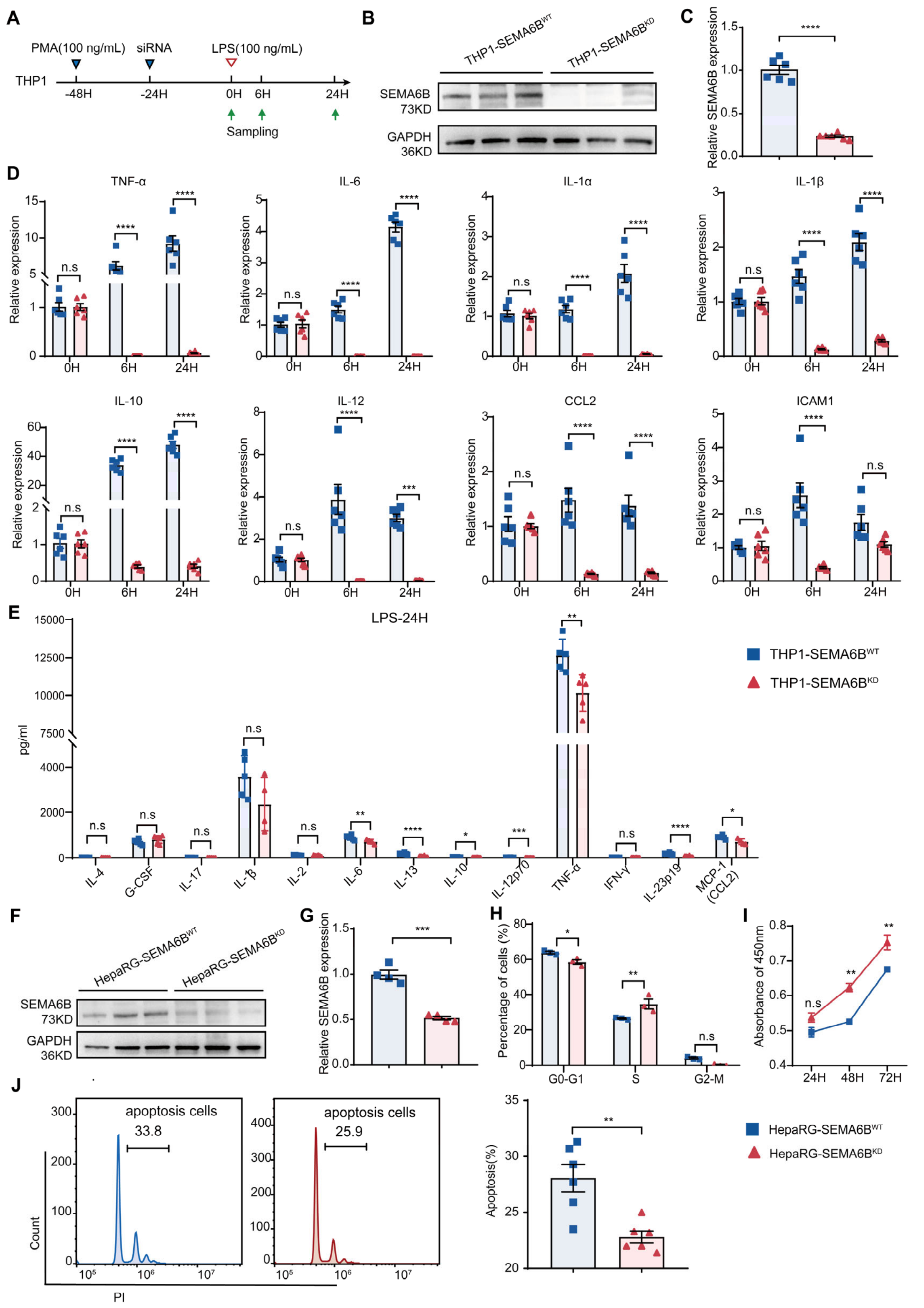

Figure S6 Evidence for the functions of SEMA6B in human macrophages and hepatocytes. (A) Schematic design for stimulation of THP1 cells. (B) Western blot and (C) qRT-PCR showing the SEMA6B expression levels in the THP1-SEMA6B^{WT} and

THP1-SEMA6B^{KD} groups. (D) Expression levels of inflammatory cytokines in the THP1-SEMA6B^{WT} and THP1-SEMA6B^{KD} groups at 0 h, 6 h, and 24 h after LPS stimulation. (E) Levels of inflammatory cytokines in the supernatants of THP1-SEMA6B^{WT} and THP1-SEMA6B^{KD} groups at 24 h after LPS stimulation. (F) Western blot and (G) qRT-PCR showing SEMA6B expression levels in the HepaRG-SEMA6B^{WT} and HepaRG-SEMA6B^{KD} groups. (H) Cytometric analysis of the cell cycle in the two groups at 24 h after LPS stimulation. (I) CCK-8 assay for cell proliferation in the two groups at 24 h, 48 h, and 72 h after LPS stimulation. (J) Cytometric analysis of apoptosis in the two groups at 24 h after LPS stimulation. Data are presented as mean ± SEM. Statistical significance: ns: not significant, *$p < 0.05$, **$p < 0.01$, ***$p < 0.001$, ****$p < 0.0001$, n = 4-6/group. KD: knockdown; WT: wild-type; LPS: lipopolysaccharide; IL: interleukin; CCL: C-C motif chemokine ligand; ICAM: intercellular cell adhesion molecule.

SUPPLEMENTARY TABLES

Table S1 Spearman correlation analysis between SEMA6B expression and clinical indexes

clinical indexes	R^2	P value
ALB	0.1270954	0.3791
ALT	0.41093	0.00303**
AST	0.2282737	0.1108
ALP	0.2194202	0.1258
TB	0.0109964	0.9396
GGT	0.1777223	0.2169
CRE	0.08728863	0.5467
Na	0.08500969	0.5572
WBC	0.1157624	0.4234
Hb	0.246988	0.0871
PCA	0.298755	0.03705*
PLT	0.1811456	0.2129
INR	0.3351427	0.02*
AFP	-0.01522282	0.9164

*p<0.05，** p <0.001

Table S2 List of protein co-interacting with SEMA6B

Protein ID				
E9QAT4	P62264	Q61545	P57776	Q8BXK4
P20152	P99024	Q8BG05	P62245	Q8BYH0
Q62167	Q60865	Q8VEK3	P62274	Q8C3W1
P20029	Q61249	Q9CXW4	P62281	Q8CCS6
P58058	Q9D1M0	Q9CZX8	P62900	Q8CDG5
P63017	Q9DBR0	Q9D662	P63101	Q8CGR7
P61979	P97461	Q8BQ46	P84089	Q8CI61
Q8BGD9	O35490	Q8CGP6	P99027	Q8JZQ9
P52480	O54951	D3YZV8	P99028	Q91W64
Q80X50	O88569	E9PY46	Q02257	Q99020
O35887	P05064	E9QAF0	Q3TIV5	Q99MR6
P60335	P06151	O88543	Q3U3V8	Q9CQE2
P63260	P10605	P05202	Q3UG20	Q9D1L0
Q61792	Q9D2U9	P09411	Q3UV17	Q9D358
Q9Z2X1	P11680	P62984	Q3V1U8	Q9D3U0
P05213	P14131	P10076	Q5DTX6	Q9EPJ9
P62082	P14206	P17095	Q60749	Q9ER73
P62908	P15864	P17182	Q61171	Q9JLC8
P63276	P16858	P23506	Q61937	Q9JMB0
P97351	P18760	P25444	Q69ZF3	Q9QXD8
P63085	P32233	P26350	Q6PD05	Q9WUU8
Q9CY58	P51410	P29341	Q6ZWR6	Q9Z0U1
P22893	P53996	P30999	Q6ZWY3	Q9Z110

O35737	P62301	P32067	Q7M725	Q9Z1D1
O54962	P62702	P35564	Q80VP2	Q9Z204
P09405	P62806	P38647	Q8BFZ3	Q9Z2U1
P10126	P62852	P41438	Q8BI67	Q9Z321
P11499	P63325	P43275	Q8BMB3	A0A0G2JDW1
E9QAT4	P68372	P56959	Q8BP92	E9Q2T3
Q792Z1	Q9CPN9			

Table S3: Antibodies used for IHC and WB

Antibodies	RRID	Company/ Cat Numb	Dilution
**Primary antibodies**			
SEMA6B	AB_2783522	Santa Cruz Biotechnology/ Cat# sc-390928	1:50/1:500
CD86	AB_869050	Abcam/ Cat# ab53004	1:1000
ALB	AB_2673704	Sigma-Aldrich/ Cat# HPA031025	1:2500
**Secondary antibodies**			
Goat anti-rabbit IgG (HRP)	AB_955447	Abcam/ Cat# ab6721	1:1000
Goat anti-mouse IgG (HRP)	AB_955439	Abcam/ Cat# ab6789	1:1000
Goat anti-rabbit IgG (H+L), CF™ 647	AB_2714032	Abcam/ Cat# ab150083	1:200
Goat anti-mouse IgG (H+L), AF™ 488		Invitrogen/ A1101	1:200

Table S4: Primers used in this study

Name		Sequence Gene	ID
mGAPDH	Forward	5’-CATCACTGCCACCCAGAAGACTG-3’	14433
	Reverse	5’-ATGCCAGTGAGCTTCCCGTTCAG-3’	
mSEMA6B	Forward	5’-CGCTGTCTTCTCAACTCCTAGC-3’	20359
	Reverse	5’-TCTTCAGGCACTGGTGTCCAGA-3’	
mIL-6	Forward	5’-TACCACTTCACAAGTCGGAGGC-3’	16193
	Reverse	5’-CTGCAAGTGCATCATCGTTGTTC-3’	
mIL-10	Forward	5’-CGGGAAGACAATAACTGCACCC-3’	16153
	Reverse	5’-CGGTTAGCAGTATGTTGTCCAGC-3’	
mIL-1β	Forward	5’-TGGACCTTCCAGGATGAGGACA-3’	16176
	Reverse	5’-GTTCATCTCGGAGCCTGTAGTG-3’	
mIL-1a	Forward	5’-ACGGCTGAGTTTCAGTGAGACC-3’	16175
	Reverse	5’-CACTCTGGTAGGTGTAAGGTGC-3’	
mTNF-a	Forward	5’-GGTGCCTATGTCTCAGCCTCTT-3’	21926
	Reverse	5’-GCCATAGAACTGATGAGAGGGAG-3’	
hSEMA6B	Forward	5’- GTCGGAGACAACATCAGCGGTA-3’	10501
	Reverse	5’-GCATCAATGGCTAGGAAGTCGG-3’	
hGAPDH	Forward	5’- CTCTCTGCTCCTCCTGTTCG-3’	2597
	Reverse	5’- ACGACCAAATCCGTTGACTC-3’	
sgRNA-mSEMA6B		5’-CACCGCACACTGTAATTGGCGCAGA-3’	
		5’-AAAC TCTGCGCCAATTACAGTGTGC-3’	
hIL-1β	Forward	5’-ATGATGGCTTATTACAGTGGCAA-3’	3553
	Reverse	5’-GTCGGAGATTCGTAGCTGGA-3’	

hIL-6	Forward	5’-ACTCACCTCTTCAGAACGAATTG-3’	3569
	Reverse	5’-CCATCTTTGGAAGGTTCAGGTTG-3’	
hIL-10	Forward	5’-GACTTTAAGGGTTACCTGGGTTG-3’	3586
	Reverse	5’-TCACATGCGCCTTGATGTCTG-3’	
hIL-1α	Forward	5’-AGATGCCTGAGATACCCAAAACC -3’	3552
	Reverse	5’-CCAAGCACACCCAGTAGTCT-3’	
hTNF-α	Forward	5’-GAGGCCAAGCCCTGGTATG -3’	7124
	Reverse	5’-CGGGCCGATTGATCTCAGC-3’	
hIL-2	Forward	5’-TACAAGAACCCGAAACTGACTCG-3’	3558
	Reverse	5’-ACATGAAGGTAGTCTCACTGCC-3’	
mCCL2	Forward	5’-TTAAAAACCTGGATCGGAACCAA-3’	20296
	Reverse	5’-GCATTAGCTTCAGATTTACGGGT-3’	
hCCL2	Forward	5’-CAGCCAGATGCAATCAATGCC-3’	6347
	Reverse	5’-TGGAATCCTGAACCCACTTCT-3’	
hICAM1	Forward	5’-ATGCCCAGACATCTGTGTCC-3’	3383
	Reverse	5’-GGGGTCTCTATGCCCAACAA-3’	
mICAM1	Forward	5’-GTGATGCTCAGGTATCCATCCA-3’	15894
	Reverse	5’-CACAGTTCTCAAAGCACAGCG-3’	
mIL-12	Forward	5’-TGGTTTGCCATCGTTTTGCTG-3’	16160
	Reverse	5’-ACAGGTGAGGTTCACTGTTTCT-3’	
hIL-12	Forward	5’-ACCCTGACCATCCAAGTCAAA-3’	3593
	Reverse	5’-TTGGCCTCGCATCTTAGAAAG-3’	
msiRNA-SEMA6B		5’-CACACUUCUACUUCAACGUTT-3’	
		5’-ACGUUGAAGUAGAAGUGUGTT-3’	

hsiRNA-SEMA6B	SEMA6B Human Pre-designed siRNA Set A (siRNA-1) (MCE, HY-RS12666)

NOTE. h, human; m, mouse.
